# Comparison of various insulin resistance surrogates on prognostic prediction and stratification following percutaneous coronary intervention in patients with and without type 2 diabetes mellitus

**DOI:** 10.1186/s12933-021-01383-7

**Published:** 2021-09-18

**Authors:** Qi Zhao, Yu-Jing Cheng, Ying-Kai Xu, Zi-Wei Zhao, Chi Liu, Tie-Nan Sun, Yu-Jie Zhou

**Affiliations:** Department of Cardiology, Beijing Anzhen Hospital, Capital Medical University, Beijing Institute of Heart Lung and Blood Vessel Disease, Beijing Key Laboratory of Precision Medicine of Coronary Atherosclerotic Disease, Clinical Center for Coronary Heart Disease, Capital Medical University, Beijing, 100029 China

**Keywords:** Insulin resistance surrogates, Type 2 diabetes mellitus, Non-ST-segment elevation acute coronary syndrome, Percutaneous coronary intervention, Major adverse cardiac and cerebrovascular events

## Abstract

**Background:**

Insulin resistance (IR), evaluation of which is difficult and complex, is closely associated with cardiovascular disease. Recently, various IR surrogates have been proposed and proved to be highly correlated with IR assessed by the gold standard. It remains indistinct whether different IR surrogates perform equivalently on prognostic prediction and stratification following percutaneous coronary intervention (PCI) in non-ST-segment elevation acute coronary syndrome (NSTE-ACS) patients with and without type 2 diabetes mellitus (T2DM).

**Methods:**

The present study recruited patients who were diagnosed with NSTE-ACS and successfully underwent PCI. IR surrogates evaluated in the current study included triglyceride-glucose (TyG) index, visceral adiposity index, Chinese visceral adiposity index, lipid accumulation product, and triglyceride-to-high density lipoprotein cholesterol ratio, calculations of which were conformed to previous studies. The observational endpoint was defined as the major adverse cardiovascular and cerebrovascular events (MACCE), including cardiac death, non-fatal myocardial infarction, and non-fatal ischemic stroke.

**Results:**

2107 patients (60.02 ± 9.03 years, 28.0% female) were ultimately enrolled in the present study. A total of 187 (8.9%) MACCEs were documented during the 24-month follow-up. Despite regarding the lower median as reference [hazard ratio (HR) 3.805, 95% confidence interval (CI) 2.581–5.608, P < 0.001] or evaluating 1 normalized unit increase (HR 1.847, 95% CI 1.564–2.181, P < 0.001), the TyG index remained the strongest risk predictor for MACCE, independent of confounding factors. The TyG index showed the most powerful diagnostic value for MACCE with the highest area under the receiver operating characteristic curve of 0.715. The addition of the TyG index, compared with other IR surrogates, exhibited the maximum enhancement on risk stratification for MACCE on the basis of a baseline model (Harrell’s C-index: 0.708 for baseline model vs. 0.758 for baseline model + TyG index, P < 0.001; continuous net reclassification improvement: 0.255, P < 0.001; integrated discrimination improvement: 0.033, P < 0.001). The results were consistent in subgroup analysis where similar analyses were performed in patients with and without T2DM, respectively.

**Conclusion:**

The TyG index, which is most strongly associated with the risk of MACCE, can be served as the most valuable IR surrogate for risk prediction and stratification in NSTE-ACS patients receiving PCI, with and without T2DM.

**Supplementary Information:**

The online version contains supplementary material available at 10.1186/s12933-021-01383-7.

## Background

Insulin resistance (IR), the most important pathogenesis for type 2 diabetes mellitus (T2DM) and metabolic syndrome, has been demonstrated to be closely related to the occurrence, progression, and prognosis of atherosclerotic cardiovascular disease (ASCVD), regardless of the presence of diabetes mellitus [1–6]. Therefore, there is undisputedly a demand for precise and prompt quantification of IR, with the aim of early identification of patients at high risk of ASCVD, assessment of disease progression, and risk stratification for adverse outcomes.

The hyperinsulinaemic-euglycaemic (HIEG) clamp, which is the gold standard technique for the evaluation of IR, has been demonstrated to be closely associated with ASCVD by previous studies [7, 8]. However, the defects of operational complexity, time consumption, and expensiveness confined it from extensive clinical application. It has been revealed that IR usually manifests as hyperglycemia, hyperinsulinemia, dyslipidemia, and central obesity (especially increased visceral fat) [6, 9]. Based on the characteristics mentioned above, various surrogate markers calculated from common laboratory and anthropometric parameters, for example, triglyceride-glucose index (TyG index), visceral adiposity index (VAI), Chinese visceral adiposity index (CVAI), lipid accumulation product (LAP), and triglyceride-to-high density lipoprotein cholesterol ratio (TG/HDL-C), have been established to alternatively evaluate the extent of IR and shown to be closely correlated with HIEG clamp [10–16]. The level of IR assessed by these surrogates has been shown in numerous studies to be significantly associated with the risk of prediabetes/diabetes, atherosclerosis, ASCVD, and adverse prognosis [17–26].

At present, comprehensive evaluation and comparison of various IR surrogates for risk prediction and stratification of adverse prognosis after percutaneous coronary intervention (PCI) in non-ST-segment elevation acute coronary syndrome (NSTE-ACS) patients with and without T2DM are still inadequate. Therefore, the current study was designed to explore the underlying relationship of various IR surrogates with adverse prognosis in this selected high-risk population, and determine the superiority among them on prognostic prediction and stratification.

## Methods

### Study population

**Fig. 1:**
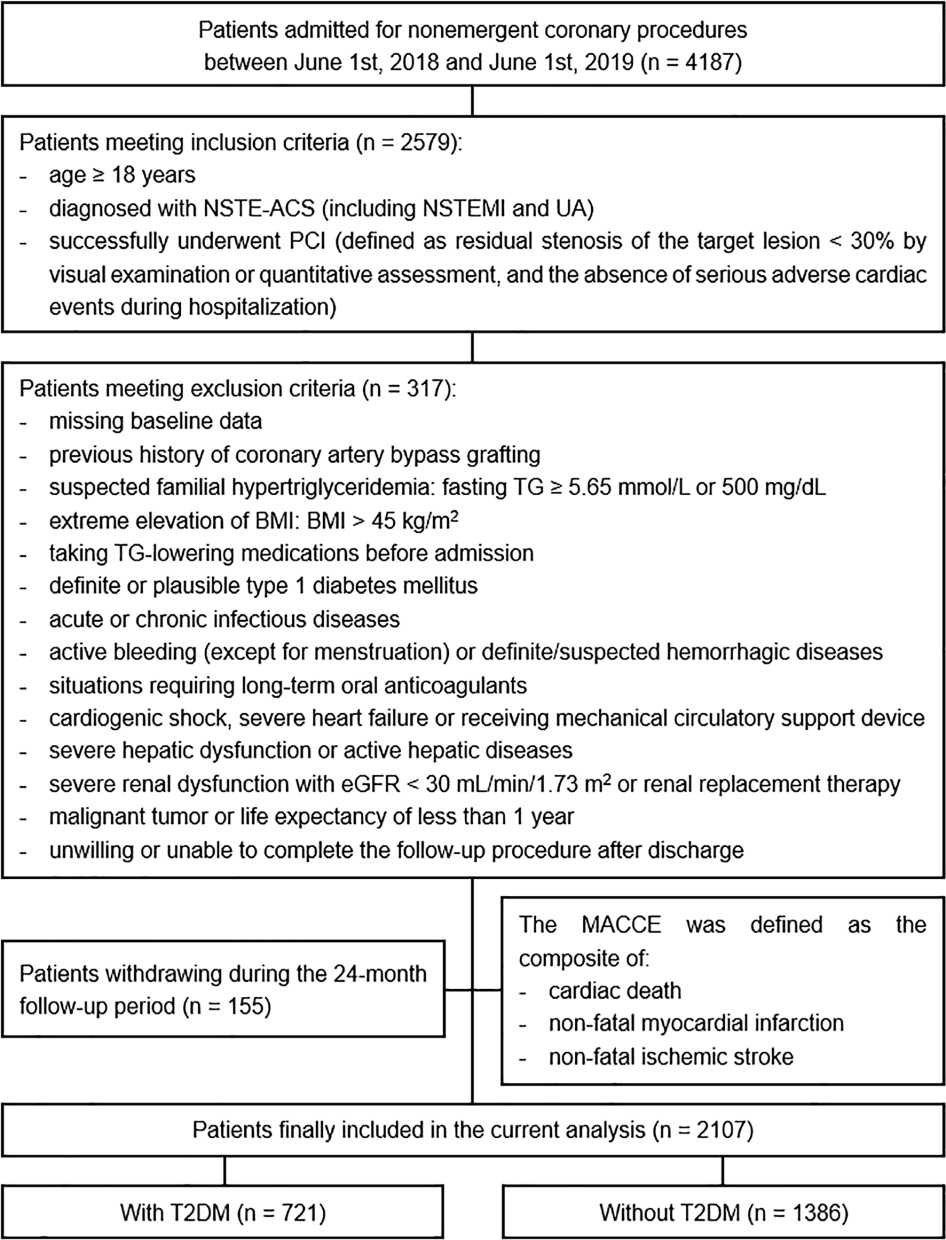
Flow diagram for the enrollment of study population. *NSTE-ACS* non-ST-segment elevation acute coronary syndrome, *NSTEMI* non-ST-segment elevation myocardial infarction, *UA* unstable angina, *PCI* percutaneous coronary intervention, *TG* triglyceride, *BMI* body mass index, *eGFR* estimated glomerular filtration rate, *MACCE* major adverse cardiac and cerebrovascular events, *T2DM* type 2 diabetes mellitus

As a single-center observational cohort study, we screened patients admitted for nonemergent coronary procedures at Beijing Anzhen Hospital, Capital Medical University, between June 1st, 2018 and June 1st, 2019. The inclusion criteria were as follows: (1) age ≥ 18 years; (2) diagnosed with NSTE-ACS [non-ST-segment elevation myocardial infarction (NSTEMI) or unstable angina (UA)], diagnostic criteria of which were referred to relevant guidelines [27]; (3) successfully underwent PCI, which was defined as residual stenosis of the target lesion < 30% by visual examination or quantitative assessment, and the absence of serious adverse cardiac events during hospitalization. Patients with missing baseline data, previous history of coronary artery bypass grafting, suspected familial hypertriglyceridemia, and/or other exclusion criteria were excluded (details shown in Fig. 1).

The study protocol was endorsed by the Clinical Research Ethics Committee of Beijing Anzhen Hospital, Capital Medical University. All subjects were informed and agreed to participate in the present study.

### Data collection and definitions

Demographic, anthropometric, laboratory, medical and procedural information was acquired by referring to the electronic medical record management system of Beijing Anzhen Hospital and then entered into an established database by trained personnel who was blinded to the study protocol.

Body mass index (BMI) was calculated as weight (kg)/[height (m)]^2^. Waist circumference (WC) was defined as the horizontal girth through the center of the umbilical, measured by using a soft ruler at the end of exhalation and before the beginning of inspiration. Patients who kept smoking at the time of admission or had quit smoking for less than 1 year and drank ≥ 12 times over the past year were considered to have a history of smoking and drinking, respectively. Patients with at least one first-degree family member having coronary artery disease (CAD) were considered to have a family history of CAD. Patients with hypertension were defined as those with previous definite diagnosis or having systolic/diastolic blood pressure ≥ 140/90 mmHg more than two times on different days during the baseline hospitalization. Patients with T2DM were defined as those with previous definite diagnosis or newly confirmed T2DM based on the practical guidelines [28]. Previous medical history of myocardial infarction (MI), PCI, stroke, and peripheral artery disease (PAD) was obtained from self-reported information and then confirmed by relevant medical records. Stroke included cerebral infarction and transient ischemic attack. PAD was defined as the artery disease that happened other than the aorta and coronary arteries with stenosis ≥ 50% and associated ischemic symptoms and/or signs.

Laboratory indices, including lipid profiles [triglyceride (TG), total cholesterol (TC), low-density lipoprotein cholesterol (LDL-C), and high-density lipoprotein cholesterol (HDL-C)], high-sensitivity C-reactive protein (hs-CRP), Creatinine, estimated glomerular filtration rate (eGFR), uric acid, and glycemic parameters [fasting blood glucose (FBG) and glycosylated hemoglobin A1c (HbA1c)], were examined with standard techniques at the core laboratory by using peripheral venous blood samples extracted in the case of fasting ≥ 8 h before coronary procedures. Left ventricular ejection fraction (LVEF) was estimated by echocardiography with the modified Simpson rule.

Medications including angiotensin-converting enzyme inhibitor (ACEI)/angiotensin receptor blocker (ARB), antiplatelet therapy, β-blocker, statins, oral antidiabetic agents, and insulin were all prescribed referring to the recommendations of practice guidelines [27] and at the discretion of the chief physicians who were unaware of the study protocol.

Coronary procedures including coronary angiography and PCI were performed by interventional cardiologists who were blind to the study protocol, in line with present guidelines in China [29]. Coronary procedural information was interpreted and recorded by two independent and experienced cardiologists who were unaware of the study protocol. Conflicts confronted during the interpretation of coronary procedures were resolved by turning to a third experienced cardiologist. Coronary lesion characteristics were described in compliance with corresponding guidelines [30]. The synergy between PCI with taxus and cardiac surgery (SYNTAX) score was determined by an online calculator (www.syntaxscore.com) to evaluate the coronary lesion complexity. Complete revascularization was defined as successful interventional procedures (residual stenosis ≤ 30%) in all coronary lesions with reference diameter ≥ 1.5 mm and stenosis ≥ 50%.

### Calculation of IR surrogates

The formulas for calculation of various IR surrogates and the cut-off value for identifying IR in previous studies were listed as follows:

**Table:** 

IR surrogates	Formulas	Cut-off value	References
TyG index	Ln [fasting TG (mg/dL) × FBG (mg/dL)/2]	4.68	[10]
VAI	Male: [WC (cm)/(39.68 + 1.88 × BMI)] × [fasting TG (mmol/L)/1.03] × [1.31/fasting HDL-C (mmol/L)] Female: [WC (cm)/(36.58 + 1.89 × BMI)] × [fasting TG (mmol/L)/0.81] × [1.52/fasting HDL-C (mmol/L)]	1.65	[11, 31]
CVAI	Male: − 267.93 + 0.68 × age + 0.03 × BMI + 4.00 × WC (cm) + 22.00 × Lg [fasting TG (mmol/L)] − 16.32 × fasting HDL-C (mmol/L) Female: − 187.32 + 1.71 × age + 4.32 × BMI + 1.12 × WC (cm) + 39.76 × Lg [fasting TG (mmol/L)] − 11.66 × fasting HDL-C (mmol/L)	Not applicable	[12]
LAP	Male: [WC (cm) − 65] × [fasting TG (mmol/L)] Female: [WC (cm) − 58] × [fasting TG (mmol/L)]	42.5	[13, 31]
TG/HDL-C	Fasting TG (mg/dL) / fasting HDL-C (mg/dL)	3.50	[14]

### Follow-up and endpoint

Patients who met the inclusion and exclusion criteria were then routinely followed up every 3 months after discharge, via telephone or outpatient service. All patients were followed up for 24 months unless withdrawal or death occurred. The observational endpoint of the present study was the major adverse cardiovascular and cerebrovascular events (MACCE), which was defined as the composite of cardiac death, non-fatal MI, and non-fatal ischemic stroke. All events were documented and further verified by referring to relevant medical records if indistinct information was acquired. The MACCE was considered to be the first adverse event that occurred during each patient's follow-up.

### Statistical analysis

Continuous variates were described as mean with standard deviation or median with interquartile range, and the comparison between two groups was examined by T-test or Mann–Whitney U-test correspondingly. Nominal variates were described as number with percentage and the comparison between two groups was examined by Chi-square test (with or without continuity correction) or Fisher’s exact test accordingly.

Cumulative no MACCE survival rates according to the median of each IR surrogate were evaluated by Kaplan–Meier analysis, and the differences between higher and lower median groups were detected by log-rank test. Unadjusted and fully adjusted Cox regression analyses were performed to evaluate the value of each IR surrogate on the prediction of MACCE. The model used in fully adjusted Cox regression analysis included smoking history, hypertension, T2DM, previous MI, previous PCI, previous stroke, clinical diagnosis, TC, hs-CRP, eGFR, HbA1c, LVEF, ACEI/ ARB at discharge, oral antidiabetic agents at discharge, insulin at discharge, left main artery (LM) disease, three-vessel disease, chronic total occlusion, SYNTAX score, complete revascularization, and number of stents. The variates were selected based on univariate analysis (P < 0.05) (Additional file 1: Table S1) and clinical experience. As determinants of IR surrogates, age, gender, BMI, WC, TG, HDL-C, and FBG were not included. The hazard ratio (HR) and 95% confidence interval (CI) for MACCE were examined by taking each IR surrogate as a nominal and continuous variate, respectively. When being taken as a nominal variate, the HR was examined by regarding the lower median of each IR surrogate as the reference. When being taken as a continuous variate, each IR surrogate was normalized by the Z-score method to compare the predictive value of them intuitively, then the HR was examined by evaluating 1 normalized unit increase. To identify the effects of medications including statins, oral antidiabetic agents, and insulin on the predictive value of IR surrogates for MACCE, sensitivity analysis was undertaken by stratifying the study population according to whether or not they were taking these medications at admission. Furthermore, the continuous relationship (linear or non-linear) between each IR surrogate and the risk of MACCE was illustrated by restricted cubic spline and examined by the likelihood ratio test.

The diagnostic value of each IR surrogate for MACCE was assessed by receiver operating characteristic (ROC) analysis. The area under the ROC curves (AUCs) were determined and then compared by the Z-test. Moreover, Harrell’s C-index, continuous net reclassification improvement (NRI), and integrated discrimination improvement (IDI) were determined to evaluate the incremental effect of each IR surrogate on risk stratification.

Similar statistical analyses described above were performed in subgroups with and without T2DM, respectively. Data analyses were performed with IBM SPSS Statistics (version 26.0), the R Programming Language (version 3.6.3), and MedCalc (version 19.1). P-value (two-tailed) < 0.05 suggested statistical significance.

## Results

Overall, 2107 patients (60.02 ± 9.03 years, 28.0% female) who met the enrollment criteria and completed the follow-up were ultimately brought into the present study. During the 24-month follow-up, 18 (0.9%) cardiac deaths, 124 (5.9%) non-fatal MIs, and 46 (2.2%) non-fatal ischemic strokes were recorded. One of the patients experienced non-fatal MI followed by non-fatal ischemic stroke. Thus, a total of 187 (8.9%) MACCEs were finally taken into the present analysis.

### Baseline characteristics of the total population

The baseline characteristics of the total population were summarized in Table 1. The levels of IR surrogates including the TyG index, VAI, CVAI, LAP, and TG/HDL-C were all significantly higher in patients with MACCE. Patients who experienced MACCE showed higher proportions of female, T2DM, hypertension, previous MI, previous PCI, and previous stroke, higher levels of WC, TG, TC, FBG, and HbA1c, while lower levels of HDL-C and LVEF. Meanwhile, more patients were diagnosed with NSTEMI and treated with ACEI/ARB, oral antidiabetic agents, and insulin in the MACCE group. As for coronary procedural information, patients in the MACCE group exhibited more LM disease, three-vessel disease, and chronic total occlusion, less complete revascularization, and higher SYNTAX score.

**Table 1:** Baseline characteristics of the total population

	Total population (n = 2107)	MACCE (n = 187)	Non-MACCE (n = 1920)	P-value
IR surrogates				
TyG index	8.87 ± 0.60	9.30 ± 0.62	8.82 ± 0.58	< 0.001
VAI	2.84 ± 1.98	3.95 ± 2.54	2.73 ± 1.88	< 0.001
CVAI	132.18 ± 45.07	151.37 ± 45.35	130.31 ± 44.61	< 0.001
LAP	51.04 ± 40.28	76.31 ± 55.99	48.58 ± 37.53	< 0.001
TG/HDL-C	4.35 ± 2.86	5.74 ± 3.51	4.21 ± 2.75	< 0.001
Age, years	60.02 ± 9.03	61.25 ± 9.89	59.90 ± 8.93	0.073
Gender, female, n (%)	591 (28.0)	65 (34.8)	526 (27.4)	0.032
BMI, kg/m^2^	26.08 ± 3.21	26.43 ± 3.30	26.05 ± 3.20	0.115
WC, cm	91.39 ± 12.39	95.24 ± 13.06	91.01 ± 12.26	< 0.001
Heart rate, bpm	69.80 ± 10.15	71.03 ± 10.40	69.68 ± 10.12	0.083
SBP, mmHg	130.17 ± 16.47	129.79 ± 17.95	130.21 ± 16.33	0.741
DBP, mmHg	76.94 ± 9.78	76.00 ± 10.26	77.03 ± 9.73	0.166
Smoking history, n (%)	1195 (56.7)	103 (55.1)	1092 (56.9)	0.636
Drinking history, n (%)	495 (23.5)	44 (23.5)	451 (23.5)	0.990
Family history of CAD, n (%)	218 (10.3)	14 (7.5)	204 (10.6)	0.179
Medical history, n (%)				
T2DM	721 (34.2)	89 (47.6)	632 (32.9)	< 0.001
Hypertension	1305 (61.9)	129 (69.0)	1176 (61.3)	0.038
Previous MI	440 (20.9)	67 (35.8)	373 (19.4)	< 0.001
Previous PCI	359 (17.0)	45 (24.1)	314 (16.4)	0.007
Previous stroke	235 (11.2)	31 (16.6)	204 (10.6)	0.014
Previous PAD	73 (3.5)	6 (3.2)	67 (3.5)	0.841
Clinical diagnosis, n (%)				0.021
NSTEMI	1750 (83.1)	43 (23.0)	314 (16.4)	
UA	357 (16.9)	144 (77.0)	1606 (83.6)	
Laboratory tests				
TG, mmol/L	1.71 ± 0.90	2.21 ± 1.07	1.66 ± 0.87	< 0.001
TC, mmol/L	4.17 ± 1.04	4.38 ± 1.11	4.14 ± 1.03	0.003
LDL-C, mmol/L	2.52 ± 0.88	2.60 ± 0.90	2.51 ± 0.88	0.208
HDL-C, mmol/L	0.99 ± 0.23	0.95 ± 0.22	0.99 ± 0.23	0.029
hs-CRP, mg/L	1.27 (0.57, 3.26)	1.46 (0.66, 3.95)	1.25 (0.55, 3.18)	0.084
Creatinine, μmol/L	75.86 ± 16.61	75.30 ± 15.85	75.91 ± 16.69	0.630
eGFR, mL/min/1.73 m^2^	93.62 ± 19.95	92.22 ± 20.40	93.75 ± 19.91	0.317
Uric acid, μmol/L	344.87 ± 80.82	351.16 ± 82.23	344.26 ± 80.67	0.265
FBG, mmol/L	6.11 ± 1.88	7.33 ± 3.08	5.99 ± 1.67	< 0.001
HbA1c, %	6.26 ± 1.18	6.90 ± 1.49	6.19 ± 1.13	< 0.001
LVEF, %	64.02 ± 6.69	62.53 ± 8.04	64.16 ± 6.53	0.008
Medications at admission, n (%)				
ACEI/ARB	470 (22.3)	46 (24.6)	424 (22.1)	0.430
DAPT	632 (30.0)	58 (31.0)	574 (29.9)	0.750
Aspirin	1105 (52.4)	103 (55.1)	1002 (52.2)	0.450
P2Y12 inhibitors	672 (31.9)	64 (34.2)	608 (31.7)	0.474
β-blocker	468 (22.2)	51 (27.3)	417 (21.7)	0.081
Statins	649 (30.8)	64 (34.2)	585 (30.5)	0.288
Oral antidiabetic agents	375 (17.8)	48 (25.7)	327 (17.0)	0.003
Insulin	198 (9.4)	29 (15.5)	169 (8.8)	0.003
Medications at discharge, n (%)				
ACEI/ARB	1455 (69.1)	154 (82.4)	1301 (67.8)	< 0.001
DAPT	2106 (100.0)	187 (100.0)	1919 (99.9)	> 0.999
Aspirin	2106 (100.0)	187 (100.0)	1919 (99.9)	> 0.999
P2Y12 inhibitors	2107 (100.0)	187 (100.0)	1920 (100.0)	1.000
β-blocker	1910 (90.7)	171 (91.4)	1739 (90.6)	0.696
Statins	2065 (98.0)	183 (97.9)	1882 (98.0)	0.881
Oral antidiabetic agents	372 (17.7)	47 (25.1)	325 (16.9)	0.005
Insulin	190 (9.0)	27 (14.4)	163 (8.5)	0.007
Coronary procedural information				
LM disease, n (%)	93 (4.4)	18 (9.6)	75 (3.9)	< 0.001
Three-vessel disease, n (%)	631 (29.9)	77 (41.2)	554 (28.9)	< 0.001
Chronic total occlusion, n (%)	277 (13.1)	39 (20.9)	238 (12.4)	0.001
Diffuse lesion, n (%)	508 (24.1)	55 (29.4)	453 (23.6)	0.076
Bifurcation lesion, n (%)	435 (20.6)	40 (21.4)	395 (20.6)	0.792
SYNTAX score	10.62 ± 5.46	12.84 ± 6.23	10.40 ± 5.33	< 0.001
Target vessel territory, n (%)				
LM	52 (2.5)	8 (4.3)	44 (2.3)	0.095
LAD	1379 (65.4)	115 (61.5)	1264 (65.8)	0.234
LCX	724 (34.4)	61 (32.6)	663 (34.5)	0.599
RCA	895 (42.5)	90 (48.1)	805 (41.9)	0.101
Complete revascularization, n (%)	1237 (58.7)	90 (48.1)	1147 (59.7)	0.002
Number of stents	1.98 ± 1.27	2.09 ± 1.34	1.97 ± 1.27	0.254

*IR* insulin resistance, *TyG* triglyceride-glucose, *VAI* visceral adiposity index, *CVAI* Chinese visceral adiposity index, *LAP* lipid accumulation product, *TG/HDL-C* triglyceride to high-density lipoprotein cholesterol ratio, *BMI* body mass index, *WC* waist circumference, *SBP* systolic blood pressure, *DBP* diastolic blood pressure, *CAD* coronary artery disease, *T2DM* type 2 diabetes mellitus, *MI* myocardial infarction, *PCI* percutaneous coronary intervention, *PAD* peripheral artery disease, *NSTEMI* non-ST-segment elevation myocardial infarction, *UA* unstable angina, *TG* triglyceride, *TC* total cholesterol, *LDL-C* low-density lipoprotein cholesterol, *HDL-C* high-density lipoprotein cholesterol, *hs-CRP* high-sensitivity C-reactive protein, *eGFR* estimated glomerular filtration rate, *FBG* fasting blood glucose, *HbA1c* glycosylated hemoglobin A1c, *LVEF* left ventricular ejection fraction, *ACEI* angiotensin converting enzyme inhibitor, *ARB* angiotensin receptor blocker, *DAPT* dual antiplatelet therapy, *LM* left main artery, *SYNTAX* synergy between PCI with taxus and cardiac surgery, *LAD* left anterior descending artery, *LCX* left circumflex artery, *RCA* right coronary artery, *MACCE* major adverse cardiac and cerebrovascular events

### Predictive value of IR surrogates for the risk of MACCE

**Fig. 2:**
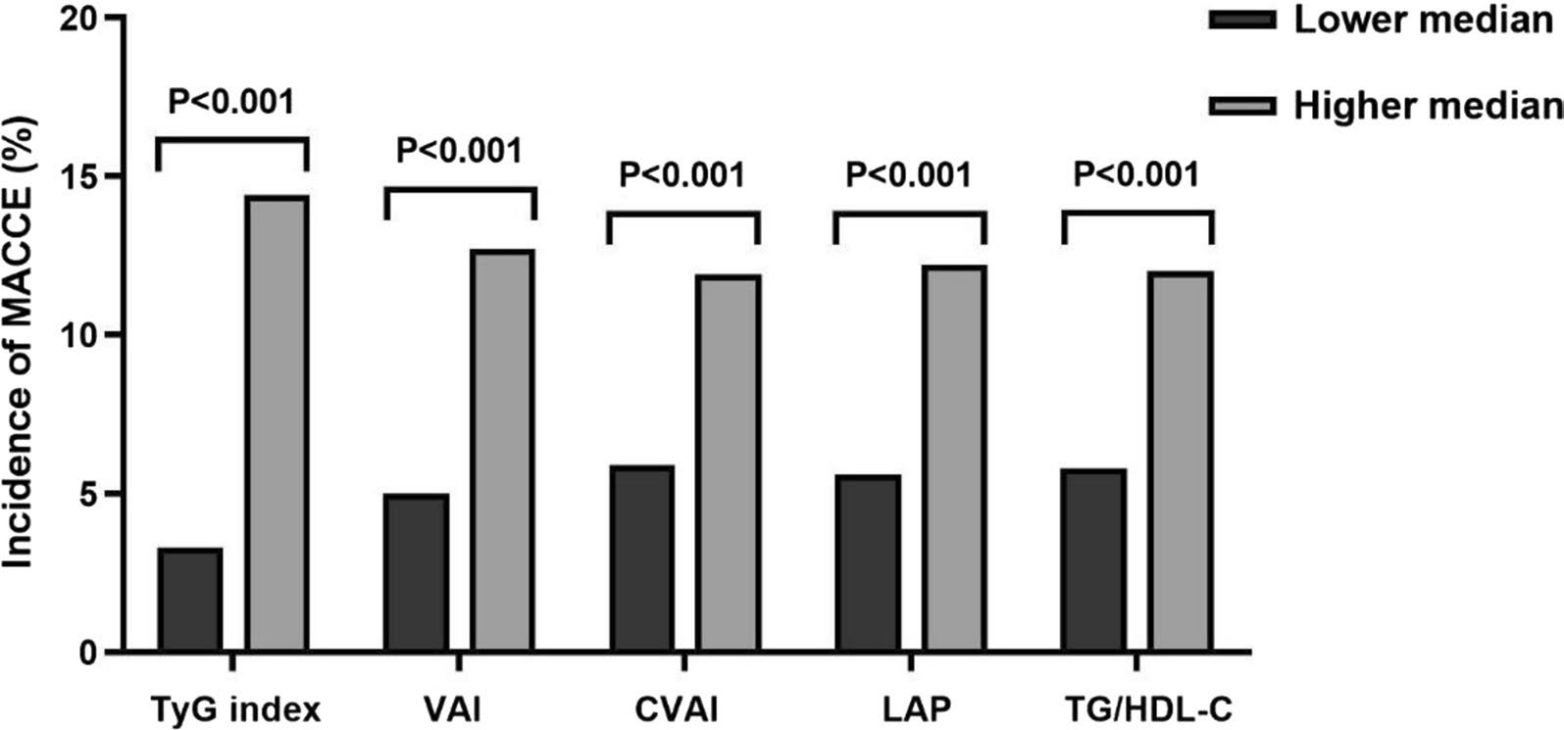
Incidence of MACCE according to the median of respective IR surrogates in the total population. *MACCE* major adverse cardiac and cerebrovascular events, *TyG* triglyceride-glucose, *VAI* visceral adiposity index, *CVAI* Chinese visceral adiposity index, *LAP* lipid accumulation product, *TG/HDL-C* triglyceride-to-high density lipoprotein cholesterol ratio

**Fig. 3:**
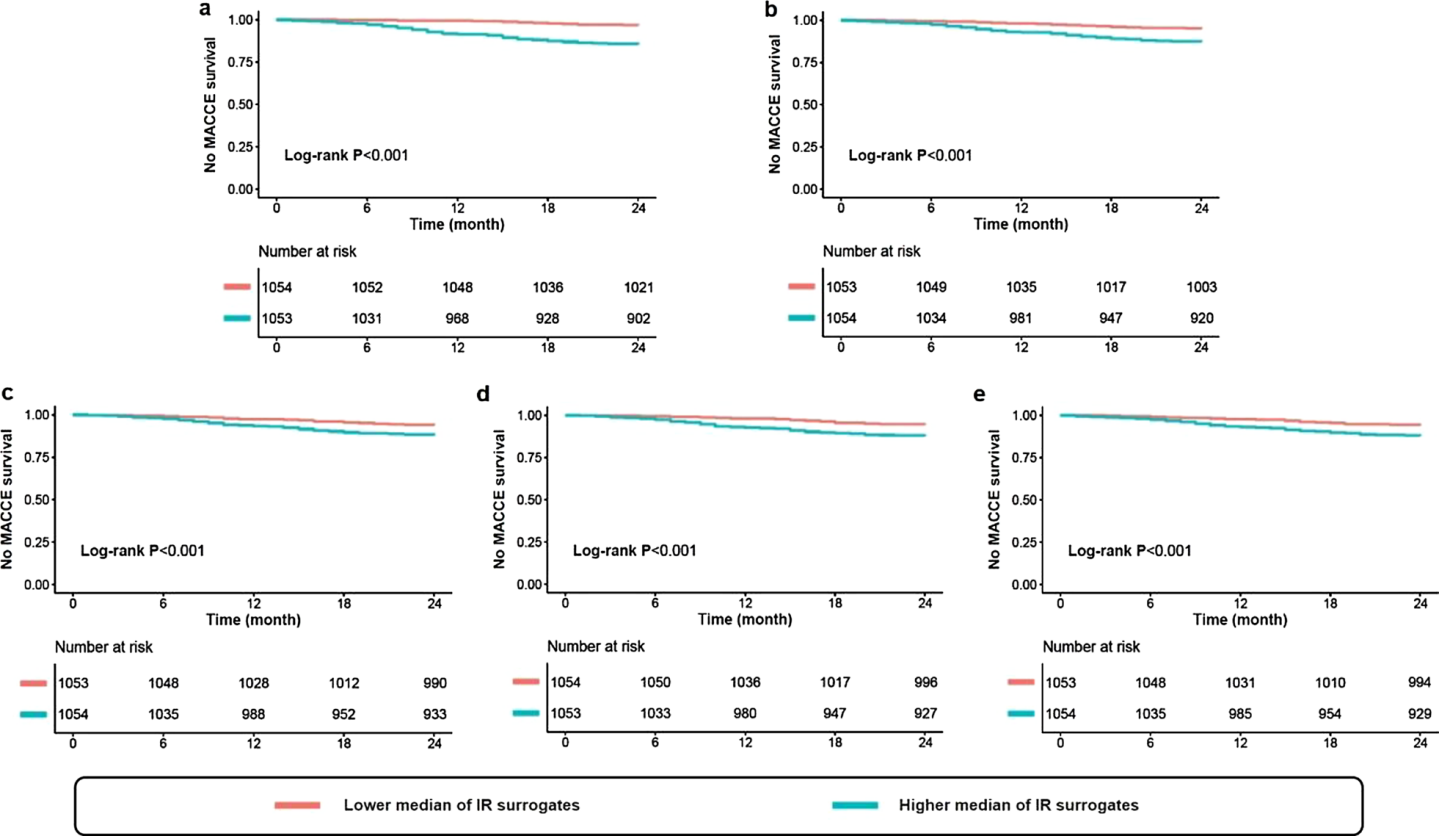
Kaplan–Meier curves for no MACCE survival according to the median of TyG index (**a**), VAI (**b**), CVAI (**c**), LAP (**d**), and TG/HDL-C (**e**) in the total population. *MACCE* major adverse cardiac and cerebrovascular events, *IR* insulin resistance

The incidence of MACCE in patients with higher median of the TyG index (14.4% vs. 3.3%), VAI (12.7% vs. 5.0%), CVAI (11.9% vs. 5.9%), LAP (12.2% vs. 5.6%), and TG/HDL-C (12.0% vs. 5.8%) was significantly higher than that in those with lower median (all P < 0.001) (Fig. 2). Meanwhile, the Kaplan–Meier analysis revealed that the patients with higher median of each IR surrogate showed significantly lower no MACCE survival rates than those with lower median (all log-rank P < 0.001) (Fig. 3).

The results of unadjusted and fully adjusted Cox regression analyses showed that the TyG index, compared with other IR surrogates, was exhibited to be the strongest risk predictor for MACCE with the highest HR, despite taking the lower median as reference (unadjusted HR 4.660, 95% CI 3.227–6.729, P < 0.001; adjusted HR 3.805, 95% CI 2.581–5.608, P < 0.001) or examining 1 normalized unit increase (unadjusted HR 2.049, 95% CI 1.793–2.343, P < 0.001; adjusted HR 1.847, 95% CI 1.564–2.181, P < 0.001). The predictive value of other IR surrogates was significant but relatively weaker than that of the TyG index (Table 2).

**Table 2:** Predictive value of various IR surrogates for the risk of MACCE in the total population

IR surrogates	Variate type	No. MACCE Lower/Higher	Unadjusted analysis		Adjusted analysis^c^	
			HR (95% CI)	P-value	HR (95% CI)	P-value
TyG index	Nominal^a^	35/152	4.660 (3.227–6.729)	< 0.001	3.805 (2.581–5.608)	< 0.001
	Continuous^b^	–	2.049 (1.793–2.343)	< 0.001	1.847 (1.564–2.181)	< 0.001
VAI	Nominal^a^	53/134	2.648 (1.926–3.639)	< 0.001	2.161 (1.559–2.996)	< 0.001
	Continuous^b^	-	1.517 (1.373–1.675)	< 0.001	1.420 (1.272–1.586)	< 0.001
CVAI	Nominal^a^	62/125	2.093 (1.544–2.838)	< 0.001	1.648 (1.203–2.258)	0.002
	Continuous^b^	-	1.533 (1.339–1.756)	< 0.001	1.353 (1.171–1.563)	< 0.001
LAP	Nominal^a^	59/128	2.273 (1.670–3.094)	< 0.001	1.764 (1.279–2.432)	0.001
	Continuous^b^	-	1.540 (1.405–1.689)	< 0.001	1.431 (1.289–1.589)	< 0.001
TG/HDL-C	Nominal^a^	61/126	2.146 (1.581–2.914)	< 0.001	1.757 (1.284–2.404)	< 0.001
	Continuous^b^	–	1.466 (1.319–1.629)	< 0.001	1.395 (1.241–1.569)	< 0.001

*IR* insulin resistance, *TyG* triglyceride-glucose, *VAI* visceral adiposity index, *CVAI* Chinese visceral adiposity index, *LAP* lipid accumulation product, *TG/HDL-C* triglyceride-to-high density lipoprotein cholesterol ratio, *MACCE* major adverse cardiac and cerebrovascular events, *HR* hazard ratio, *CI* confidence interval

^a^The HR was examined by regarding the lower median as reference

^b^The HR was examined by evaluating 1 normalized unit increase

^c^Adjusted for smoking history, hypertension, T2DM, previous MI, previous PCI, previous stroke, clinical diagnosis, TC, hs-CRP, eGFR, HbA1c, LVEF, ACEI/ARB at discharge, oral antidiabetic agents at discharge, insulin at discharge, LM disease, three-vessel disease, chronic total occlusion, SYNTAX score, complete revascularization, and number of stents

**Fig. 4:**
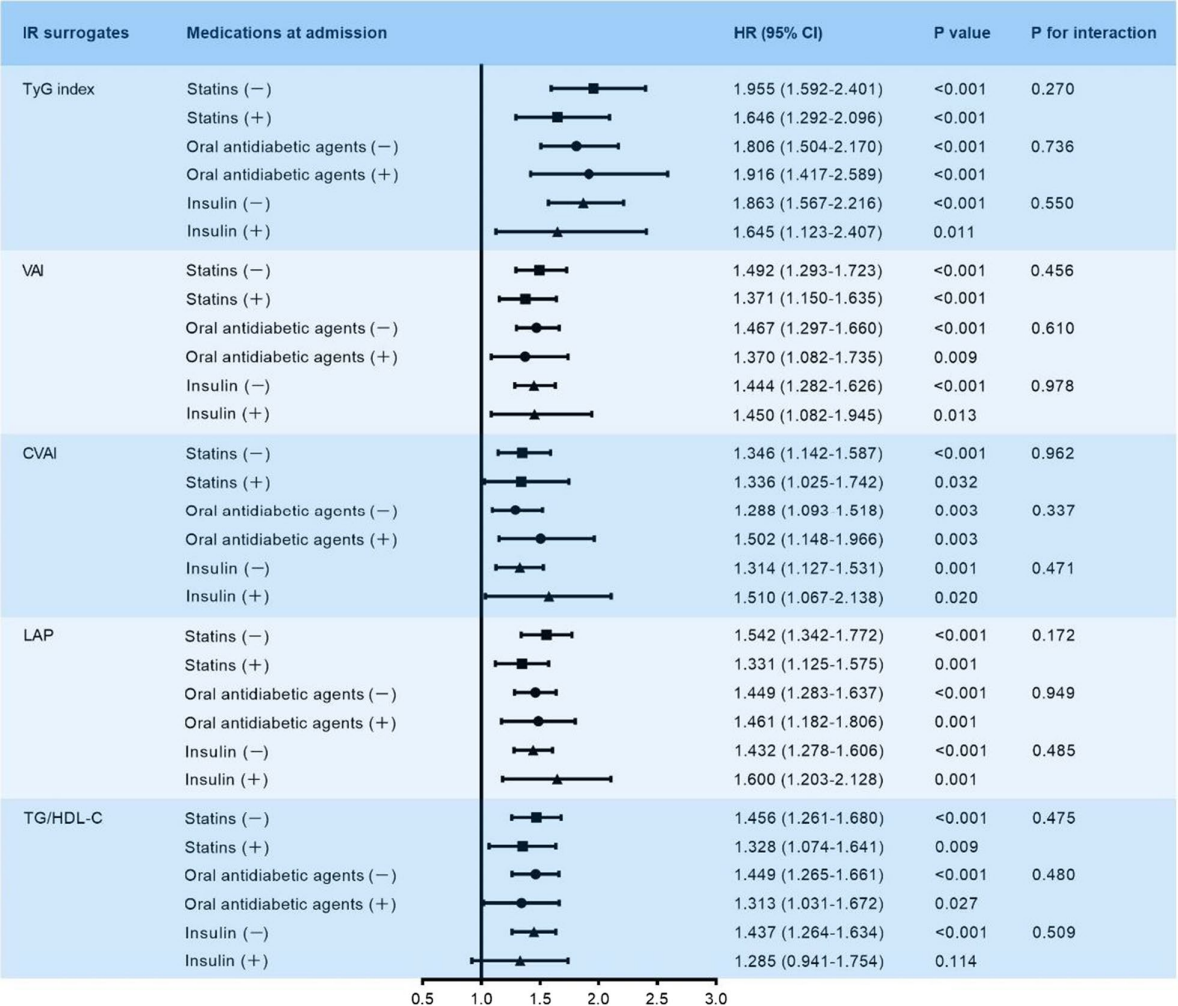
Sensitivity analysis stratified by the medications at admission. Adjusted for smoking history, hypertension, T2DM, previous MI, previous PCI, previous stroke, clinical diagnosis, TC, hs-CRP, eGFR, HbA1c, LVEF, ACEI/ARB at discharge, oral antidiabetic agents at discharge, insulin at discharge, LM disease, three-vessel disease, chronic total occlusion, SYNTAX score, complete revascularization, and number of stents. The HR was examined by evaluating 1 normalized unit increase. *IR* insulin resistance, *TyG* triglyceride-glucose, *VAI* visceral adiposity index, *CVAI* Chinese visceral adiposity index, *LAP* lipid accumulation product, *TG/HDL-C* triglyceride-to-high density lipoprotein cholesterol ratio, *HR* hazard ratio, *CI* confidence interval

**Fig. 5:**
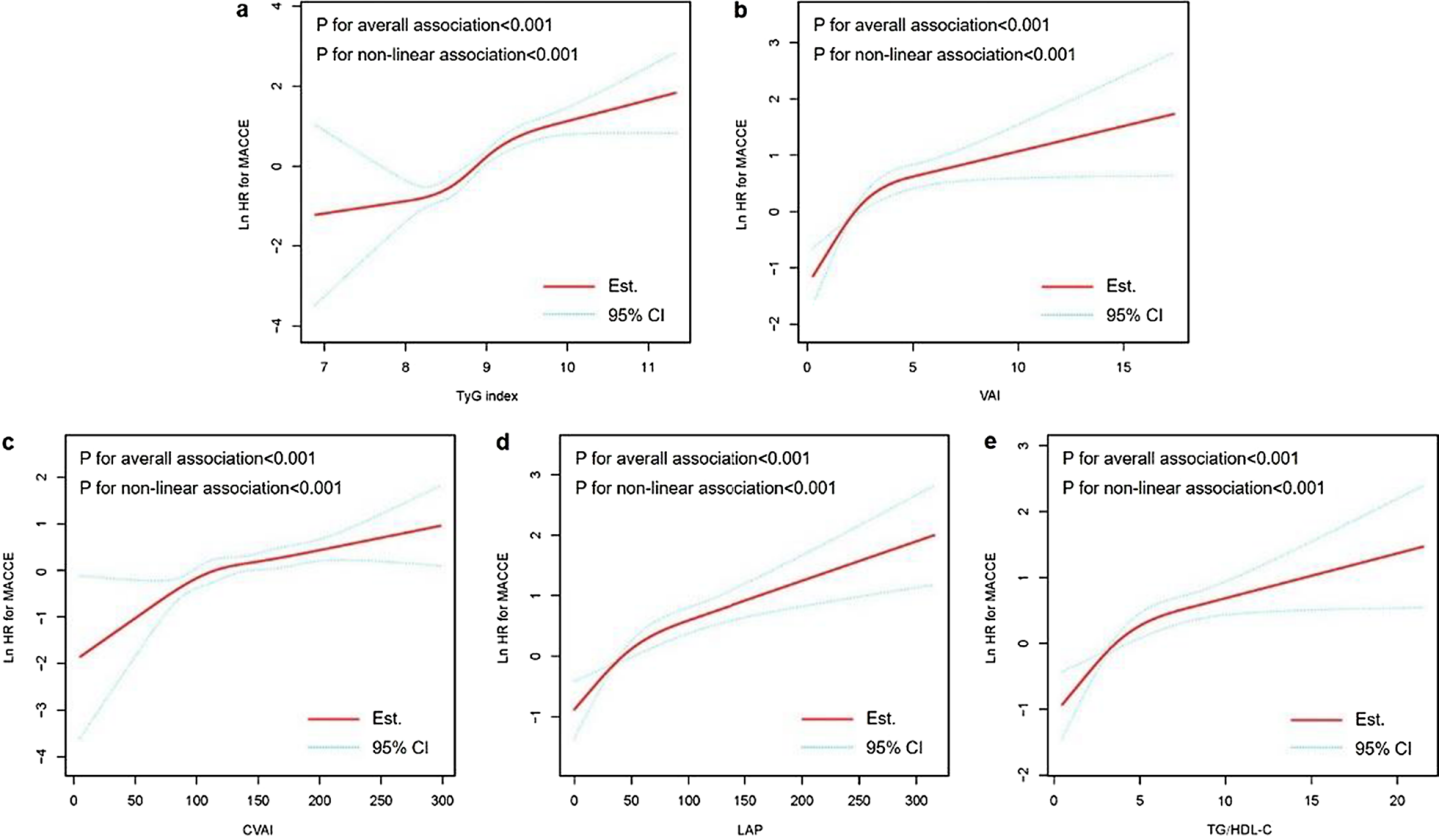
Restricted cubic splines for the risk of MACCE according to TyG index (**a**), VAI (**b**), CVAI (**c**), LAP (**d**), and TG/HDL-C (**e**). Adjusted for smoking history, hypertension, T2DM, previous MI, previous PCI, previous stroke, clinical diagnosis, TC, hs-CRP, eGFR, HbA1c, LVEF, ACEI/ARB at discharge, oral antidiabetic agents at discharge, insulin at discharge, LM disease, three-vessel disease, chronic total occlusion, SYNTAX score, complete revascularization, and number of stents. *HR* hazard ratio, *MACCE* major adverse cardiac and cerebrovascular events, *CI* confidence interval, *TyG* triglyceride-glucose, *VAI* visceral adiposity index, *CVAI* Chinese visceral adiposity index, *LAP* lipid accumulation product, *TG/HDL-C* triglyceride-to-high density lipoprotein cholesterol ratio

Since the determinants of IR surrogates may be affected by medications, especially lipid-lowering and hypoglycemic agents, sensitivity analysis was performed and revealed that each IR surrogate was robustly associated with the risk of MACCE, regardless of whether a statin, oral antidiabetic agent, or insulin was administered at admission (all P for interaction > 0.05) (Fig. 4). Moreover, restricted cubic spline analysis elucidated that there was a linear association between each IR surrogate and the risk of MACCE (all P for non-linear association < 0.001) (Fig. 5).

### Diagnostic performance of IR surrogates for MACCE

**Fig. 6:**
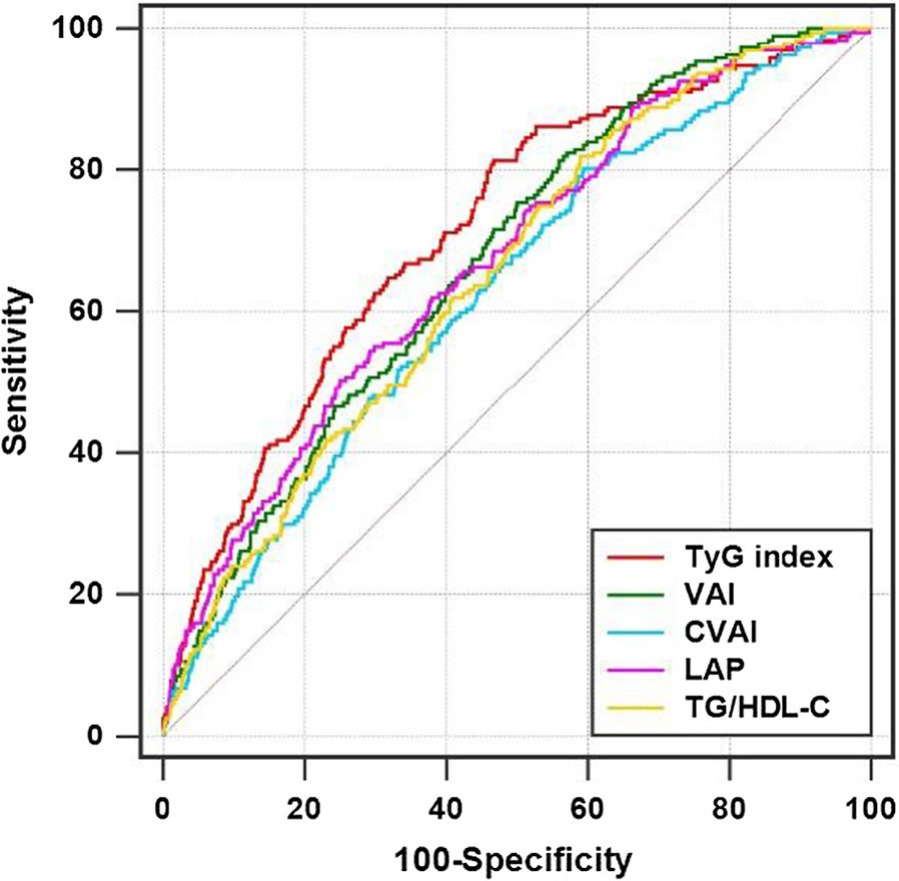
ROC curves evaluating the diagnostic performance of each IR surrogate for MACCE in the total population. *TyG* triglyceride-glucose, *VAI* visceral adiposity index, *CVAI* Chinese visceral adiposity index, *LAP* lipid accumulation product, *TG/HDL-C* triglyceride-to-high density lipoprotein cholesterol ratio

The diagnostic performance of each IR surrogate for MACCE was assessed and compared by ROC curve analysis. The TyG index (0.715, 95% CI 0.695–0.734) displayed the highest diagnostic ability manifested as the maximum AUC, in comparison with VAI (0.673, 95% CI 0.652–0.693, P for comparison = 0.001), CVAI (0.628, 95% CI 0.607–0.649, P for comparison < 0.001), LAP (0.670, 95% CI 0.650–0.691, P for comparison = 0.001), and TG/HDL-C (0.651, 95% CI 0.630–0.671, P for comparison < 0.001) (Table 3; Fig. 6). In addition, the cut-off value, sensitivity, and specificity for each IR surrogate were calculated, respectively (details shown in Table 3).

**Table 3:** Diagnostic performance of IR surrogates for MACCE in the total population

	AUC			Cut-off value	Sensitivity, %	Specificity, %
	Est. (95% CI)	P-value	P for comparison			
TyG index	0.715 (0.695–0.734)	< 0.001	–	8.85	81.28	53.13
VAI	0.673 (0.652–0.693)	< 0.001	0.001	2.20	75.40	49.84
CVAI	0.628 (0.607–0.649)	< 0.001	< 0.001	117.38	80.21	40.47
LAP	0.670 (0.650–0.691)	< 0.001	0.001	56.94	54.55	70.68
TG/HDL-C	0.651 (0.630–0.671)	< 0.001	< 0.001	3.02	81.82	40.94

*TyG* triglyceride-glucose, *VAI* visceral adiposity index, *CVAI* Chinese visceral adiposity index, *LAP* lipid accumulation product, *TG/HDL-C* triglyceride-to-high density lipoprotein cholesterol ratio, *AUC* area under the ROC curve, *CI* confidence interval

### Incremental effect of IR surrogates on risk stratification

The addition of the TyG index, in comparison with other IR surrogates, exhibited the maximum enhancement on risk stratification for MACCE on the basis of the baseline model including recognized risk factors (smoking history, hypertension, T2DM, previous MI, previous PCI, previous stroke, TC, eGFR, HbA1c, LVEF, LM disease, three-vessel disease, SYNTAX score, and number of stents), in terms of increased Harrell’s C-index (0.708, 95% CI 0.672–0.744 for baseline model vs. 0.758, 95% CI 0.726–0.791 for baseline model + TyG index, P < 0.001), and significant continuous NRI (0.255, 95% CI 0.145–0.320, P < 0.001) and IDI (0.033, 95% CI 0.012–0.058, P < 0.001). Significant but relatively minor incremental effects were obtained after adding other IR surrogates into the baseline model (Table 4).

**Table 4:** Incremental ability of various IR surrogates on the prediction of MACCE in the total population

	Harrell’s C-index			Continuous NRI		IDI	
	Est. (95% CI)	ΔEst	P-value	Est. (95% CI)	P-value	Est. (95% CI)	P-value
Baseline model^a^	0.708 (0.672–0.744)	–	–	–	–	–	–
+ TyG index	0.758 (0.726–0.791)	0.050	< 0.001	0.255 (0.145–0.320)	< 0.001	0.033 (0.012–0.058)	< 0.001
+ VAI	0.734 (0.700–0.769)	0.026	0.004	0.149 (0.076–0.244)	< 0.001	0.025 (0.010–0.046)	< 0.001
+ CVAI	0.726 (0.692–0.761)	0.018	0.029	0.168 (0.033–0.239)	0.007	0.008 (0.000–0.023)	0.033
+ LAP	0.742 (0.707–0.777)	0.034	< 0.001	0.155 (0.060–0.233)	< 0.001	0.026 (0.009–0.053)	< 0.001
+ TG/HDL-C	0.731 (0.696-.0766)	0.023	< 0.001	0.134 (0.054–0.227)	< 0.001	0.020 (0.006–0.041)	< 0.001

*TyG* triglyceride-glucose, *VAI* visceral adiposity index, *CVAI* Chinese visceral adiposity index, *LAP* lipid accumulation product, *TG/HDL-C* triglyceride-to-high density lipoprotein cholesterol ratio, *NRI* net reclassification improvement, *IDI* integrated discrimination improvement, *CI* confidence interval

^a^The baseline model incorporates smoking history, hypertension, T2DM, previous MI, previous PCI, previous stroke, TC, eGFR, HbA1c, LVEF, LM disease, three-vessel disease, SYNTAX score, and number of stents

### Subgroup analysis based on T2DM

The total population was divided into two subgroups (with T2DM, n = 721 and without T2DM, n = 1386). The incidence of MACCE in subgroups with and without T2DM was 12.3% and 7.1%, respectively. The differences in WC, previous MI, TG, FBG, HbA1c, ACEI/ARB, LM disease, and SYNTAX score between MACCE and non-MACCE group remained significant in subgroups with and without T2DM. However, the discrepancies in gender, previous PCI, TC, three-vessel disease, and chronic total occlusion were only significant in the subgroup with T2DM, while the differences in age, previous stroke, LVEF, β-blocker, complete revascularization were only significant in the subgroup without T2DM (Additional file 1: Table S2).

In both subgroups, the incidence of MACCE in patients with higher median of each IR surrogate was significantly higher than that in those with lower median (Additional file 2: Figure S1). Similar to the Kaplan–Meier analysis in the total population, the differences in no MACCE survival rates between higher and lower median of each IR surrogate remained significant in subgroups with and without T2DM (Additional file 2: Figure S2). In unadjusted and fully adjusted Cox regression analyses, the TyG index was consistently shown to have the most powerful risk prediction ability in both subgroups, despite being taken as a nominal and continuous variate. For other IR surrogates, significant but relatively weaker predictive abilities were obtained (Additional file 1: Table S3).

As for ROC curve analysis, the TyG index exhibited the highest AUC among all IR surrogates in subgroups with and without T2DM. Of note, the difference in AUCs was not significant between the TyG index and LAP in the subgroup with T2DM (Additional file 1: Table S4; Additional file 2: Figure S3). Moreover, the TyG index continued to exhibit the strongest incremental effect on risk stratification beyond the baseline model in both subgroups. Nevertheless, minor or insignificant incremental effects were acquired with the addition of other IR surrogates into the baseline model (Additional file 1: Table S5).

## Discussion

The current analyses showed that compared with other IR surrogates, the TyG index consistently showed the most powerful ability on risk prediction, despite the adjustment of confounding factors. The TyG index exhibited the strongest diagnostic value for MACCE with the highest AUC. After being introduced into the baseline model, the TyG index, in comparison with other IR surrogates, exhibited the maximum incremental effect on risk stratification for MACCE. For other IR surrogates, significant but relatively weaker abilities on risk prediction and stratification were obtained. The results were unanimous in the subgroup analysis where similar analyses were performed in patients with and without T2DM, respectively.

### Clinical significance and quantification of IR

IR, which is characterized as decreasing efficiency and compensatory secretion of insulin [9], has been well demonstrated to be closely associated with ASCVD. Since the gold standard method is complex, time-consuming, and expensive, it is of great clinical significance to explore simple surrogate markers for evaluating IR.

The homeostasis model assessment of IR (HOMA-IR), which is determined by FBG and fasting insulin, the two most important elements of IR, has been regarded as the generally accepted surrogate marker of IR and shown to be closely related to ASCVD [32]. However, the measurement of fasting insulin is not routinely conducted in clinical practice and varies between different laboratories, especially for non-diabetic patients, which makes HOMA-IR unsuitable for widespread clinical application. Besides increased FBG and fasting insulin, IR has been also proved to be characterized as dyslipidemia (especially increased fasting TG and decreased HDL-C), and visceral obesity [6, 9]. On account of these characteristics, various indicators originated from conventional laboratory and anthropometric indices have been proposed as substitutes for assessing the level of IR. Unlike the HIEG clamp and HOMA-IR, which are complicated, time and cost consuming, and insulin-dependent, IR surrogates including the TyG index, VAI, CVAI, LAP, and TG/HDL-C exhibit the superiority of simplicity, accessibility, inexpensiveness, and insulin-independence, indicating the great potential of them to be widely used in clinic as valuable indicators reflecting the level of IR.

### Associations between IR surrogates and ASCVD

IR surrogates have been proved to be closely related to the development and progression of ASCVD in subsequent studies. Irace et al. [19] found that the TyG index exhibited a higher correlation than HOMA-IR with carotid atherosclerosis, suggesting that the TyG index could better reflect the cardiovascular risk. The study of Cho et al. [33] showed that elevated TyG index and TG/HDL-C were independently correlated with an increased risk of CAD. However, the correlation was only significant in non-diabetic patients, and HOMA-IR did not show a predictive value for CAD in this study. The association of the TyG index with the risk of developing ASCVD has also been verified by a series of studies, in both diabetic and non-diabetic population [20, 34–36]. The ATTICA study [21, 22] revealed that VAI and LAP were independently associated with long-term risk of ASCVD, indicating that VAI and LAP could be useful predictors for identifying individuals at high risk of ASCVD in the general population. Findings from Da silva et al. [23] further revealed that increased TyG index was significantly associated with a higher prevalence of symptomatic CAD in patients in secondary care. In addition, for patients with pre-existing ASCVD, IR surrogates including the TyG index and TG/HDL-C have been shown to be significantly associated with the risk of adverse prognosis [24–26, 37–39].

However, there is a relative lack of researches aiming at investigating and comparing the value of various IR surrogates on the prediction and stratification for the risk of adverse prognosis following PCI in patients diagnosed with NSTE-ACS. The present study, which confirmed the prognostic impact of various IR surrogates and further identified the superiority of the TyG index over other IR surrogates on the prediction and stratification for the risk of MACCE, fills in the gaps of previous studies in the comparison of the prognostic value of different IR surrogates. Moreover, the superiority of the TyG index on risk prediction and stratification was shown to be consistent in both subgroups with and without T2DM, suggesting that the TyG index could be served as the most valuable IR surrogate providing more information on adverse prognosis, independent of the presence of T2DM.

### Potential explanations for the superiority of the TyG index on the prognostic value

As described above, IR is usually characterized as increased FBG, fasting insulin, and fasting TG, decreased HDL-C, central obesity (especially increased visceral fat), and so forth, but the roles and importance they played in the quantification of IR have not been fully investigated. IR surrogates derived from different combinations of the characteristics mentioned above may reflect the level of IR from various aspects. Former study has shown that fasting TG mainly reflects IR from the adipose tissue, whereas FBG mainly reflects IR from the liver [40]. Therefore, it can be easily generalized that the TyG index, which is calculated from fasting TG and FBG, may reflect IR from the two most significant dimensions, thus making it more closely associate with the level of IR. This may be an important part of the explanation for the results of the present study that the TyG index, in contrast to other IR surrogates, plays the most pronounced role in prediction and stratification for the risk of adverse outcomes.

In addition, a large number of studies have shown that the TyG index is closely related to vascular calcification [41–43], and arterial stiffness assessed by pulse wave velocity [44, 45], both of which were important risk predictors for ASCVD. Moreover, the association between the TyG index and a variety of risk factors for ASCVD, for example, hypertension [46], renal dysfunction [47], and hyperuricemia [48], was also confirmed by previous studies. As for other IR surrogates, however, the corresponding evidence was relatively lacking, which may be another important explanation for the superiority of the TyG index.

### Mechanisms mediating the associations between IR surrogates and ASCVD

Since the high correlation between IR surrogates and the HIEG clamp, the gold standard method for evaluating the IR levels, has been well established by former studies [10, 14–16], the close relationship of IR surrogates with ASCVD may be mainly mediated by IR, which promotes the development and progression of atherosclerosis through various mechanisms. It has been widely illustrated that IR is closely associated with endothelial dysfunction, oxidative stress, smooth muscle cell proliferation and migration, inflammatory response, and endothelin-1 overproduction [6, 49, 50], all of which have been considered to be significant pathogenesis for the formation and progression of atherosclerosis. Furthermore, IR has been also revealed to be highly correlated with cardiovascular remodeling, myocardial hypoperfusion, microcirculatory dysfunction, and thrombosis imbalance [51–53], which may be the potential explanations for the significant predictive value of IR surrogates for adverse prognosis.

### Study strengths and limitations

The current study, which investigated the predictive value of various IR surrogates for adverse prognosis in patients with NSTE-ACS who were treated with PCI, remarkably found that the TyG index showed exceptional performance beyond other IR surrogates on risk prediction and stratification in this selected high-risk population. More importantly, the superiority of the TyG index remained consistent regardless of the presence of T2DM. To our knowledge, this is the first study comprehensively exploring the superiority of different IR surrogates on risk prediction and stratification for adverse prognosis following PCI in patients with NSTE-ACS.

However, some limitations listed as follows need to be noted. (1) As a single-center, observational cohort study with a relatively shorter follow-up time, the statistical power may be limited. (2) The study population was highly selected with strict enrollment criteria, which may result in selection bias and difficulties in generalizing the findings to other populations. (3) Despite dynamic changes [54] and the mean of multiple monitoring [55] of the TyG index during the follow-up having been proved to be closely associated with cardiovascular events, they were not accessible in the current analysis. Further group-based trajectory analysis is needed to provide more reliable findings. (4) Lipid-lowering and antidiabetic therapy, though adjusted and/or alleviated in analysis, may have an underlying impact on the study results. (5) The diet characteristics, which may have a great influence on the estimation of IR surrogates, were not available in the present study. (6) Although blood samples were obtained at a fasting time of ≥ 8 h, the definite fasting duration was not accessible, which may have a potential effect on the results. (7) The gold standard and generally admitted methods for evaluating IR, HIEG clamp and HOMA-IR, were not attainable in this study, which makes the comparison between the existing IR surrogates and them unavailable.

## Conclusion

Compared with other IR surrogates, the TyG index is most strongly associated with the risk of MACCE in NSTE-ACS patients who received PCI, either in those with or without T2DM. The TyG index, which is derived from fasting TG and FBG, may provide more valuable information than other IR surrogates in identifying patients at high risk of developing adverse prognosis in this selected population.

## Supplementary Information

**Additional file 1.** Additional Tables.

**Additional file 2. ** Additional Figures.

## Data Availability

The datasets used and/or analyzed during the present study are available from the corresponding author on reasonable request.

## Abbreviations

IR: Insulin resistance
T2DM: Type 2 diabetes mellitus
ASCVD: Atherosclerotic cardiovascular disease
HIEG: Hyperinsulinaemic-euglycaemic
TyG: Triglyceride-glucose
VAI: Visceral adiposity index
CVAI: Chinese visceral adiposity index
LAP: Lipid accumulation product
TG/HDL-C: Triglyceride-to-high density lipoprotein cholesterol ratio
PCI: Percutaneous coronary intervention
NSTE-ACS: Non-ST-segment elevation acute coronary syndrome
NSTEMI: Non-ST-segment elevation myocardial infarction
UA: Unstable angina
BMI: Body mass index
WC: Waist circumference
CAD: Coronary artery disease
MI: Myocardial infarction
PAD: Peripheral artery disease
TG: Triglyceride
TC: Total cholesterol
LDL-C: Low-density lipoprotein cholesterol
HDL-C: High-density lipoprotein cholesterol
hs-CRP: High-sensitivity C-reactive protein
eGFR: Estimated glomerular filtration rate
FBG: Fasting blood glucose
HbA1c: Glycosylated hemoglobin A1c
LVEF: Left ventricular ejection fraction
ACEI: Angiotensin-converting enzyme inhibitor
ARB: Angiotensin receptor blocker
SYNTAX: The synergy between PCI with taxus and cardiac surgery
MACCE: Major cardiovascular and cerebrovascular events
LM: Left main artery
HR: Hazard ratio
CI: Confidence interval
ROC: Receiver operating characteristics
AUC: Area under the ROC curve
NRI: Net reclassification improvement
IDI: Integrated discrimination improvement
HOMA-IR: Homeostasis model assessment of insulin resistance
