# Supplementary material for: Comparison of various insulin resistance surrogates on prognostic prediction and stratification following percutaneous coronary intervention in patients with and without type 2 diabetes mellitus
Source: Cardiovasc Diabetol. 2021 Sep 18;20:190. doi: 10.1186/s12933-021-01383-7 (PMC8449896; doi:10.1186/s12933-021-01383-7)
Supplement: Supplementary file 1 — Additional file 1. Additional Tables. [file 12933_2021_1383_MOESM1_ESM.docx]

# Table S1. Univariate Cox regression analysis investigating potential predictors of MACCE

|  | MACCE | | |
| --- | --- | --- | --- |
|  | HR | 95% CI | P-value |
| Age, per 1 year | 1.016 | 1.000-1.033 | 0.057 |
| Gender, male as reference | 1.379 | 1.021-1.863 | 0.036 |
| BMI, per 1 kg/m^2^ | 1.037 | 0.993-1.084 | 0.101 |
| WC, per 1 cm | 1.026 | 1.015-1.038 | < 0.001 |
| Heart rate, per 1 bpm | 1.012 | 0.998-1.025 | 0.084 |
| SBP, per 1 mmHg | 0.998 | 0.990-1.007 | 0.736 |
| DBP, per 1 mmHg | 0.990 | 0.975-1.005 | 0.177 |
| Smoking history | 0.937 | 0.703-1.250 | 0.660 |
| Drinking history | 1.000 | 0.713-1.402 | 0.999 |
| Family history of CAD | 0.691 | 0.401-1.191 | 0.184 |
| T2DM | 1.805 | 1.355-2.405 | < 0.001 |
| Hypertension | 1.393 | 1.022-1.898 | 0.036 |
| Previous MI | 2.221 | 1.647-2.995 | < 0.001 |
| Previous PCI | 1.575 | 1.126-2.202 | 0.008 |
| Previous stroke | 1.619 | 1.101-2.381 | 0.014 |
| Previous PAD | 0.917 | 0.406-2.068 | 0.834 |
| Clinical diagnosis, UA as reference | 1.493 | 1.062-2.099 | 0.021 |
| TG, per 1 mmol/L | 1.653 | 1.461-1.870 | < 0.001 |
| TC, per 1 mmol/L | 1.216 | 1.072-1.379 | 0.002 |
| LDL-C, per 1 mmol/L | 1.110 | 0.951-1.296 | 0.184 |
| HDL-C, per 1 mmol/L | 0.484 | 0.252-0.932 | 0.030 |
| hs-CRP, per 1 mg/L | 1.012 | 0.991-1.034 | 0.263 |
| eGFR, per 1 mL/min/1.73 m^2^ | 0.997 | 0.989-1.004 | 0.357 |
| FBG, per 1 mmol/L | 1.188 | 1.148-1.230 | < 0.001 |
| HbA1c, per 1% | 1.422 | 1.303-1.553 | < 0.001 |
| LVEF, per 1% | 0.969 | 0.950-0.987 | 0.001 |
| ACEI/ARB at admission | 1.149 | 0.824-1.603 | 0.414 |
| DAPT at admission | 1.051 | 0.771-1.433 | 0.751 |
| Statins at admission | 1.167 | 0.863-1.579 | 0.316 |
| Oral antidiabetic agents at admission | 1.643 | 1.183-2.280 | 0.003 |
| Insulin at admission | 1.856 | 1.249-2.757 | 0.002 |
| ACEI/ARB at discharge | 2.166 | 1.487-3.155 | < 0.001 |
| Statins at discharge | 0.919 | 0.341-2.474 | 0.867 |
| Oral antidiabetic agents at discharge | 1.610 | 1.157-2.240 | 0.005 |
| Insulin at discharge | 1.784 | 1.187-2.683 | 0.005 |
| LM disease | 2.460 | 1.513-4.000 | < 0.001 |
| Three-vessel disease | 1.684 | 1.258-2.253 | < 0.001 |
| Chronic total occlusion | 1.830 | 1.286-2.604 | 0.001 |
| Diffuse lesion | 1.331 | 0.972-1.824 | 0.075 |
| Bifurcation lesion | 1.049 | 0.740-1.488 | 0.788 |
| SYNTAX score, per 1-point | 1.075 | 1.049-1.100 | < 0.001 |
| Complete revascularization | 0.640 | 0.480-0.852 | 0.002 |
| Number of stents, per 1 stent | 1.064 | 0.956-1.184 | 0.256 |

*BMI* body mass index, *WC* waist circumference, *SBP* systolic blood pressure, *DBP* diastolic blood pressure, *CAD* coronary artery disease, *T2DM* type 2 diabetes mellitus, *MI* myocardial infarction, *PCI* percutaneous coronary intervention, *PAD* peripheral artery disease, *UA* unstable angina, *TG* triglyceride, *TC* total cholesterol, *LDL-C* low-density lipoprotein cholesterol, *HDL-C* high-density lipoprotein cholesterol, *hs-CRP* high-sensitivity C-reactive protein, *eGFR* estimated glomerular filtration rate, *FBG* fasting blood glucose, *HbA1c* glycosylated hemoglobin A1c, *LVEF* left ventricular ejection fraction, *ACEI* angiotensin converting enzyme inhibitor, *ARB* angiotensin receptor blocker, *DAPT* dual antiplatelet therapy, *LM* left main artery, *SYNTAX* synergy between PCI with taxus and cardiac surgery

# Table S2. Baseline characteristics of subgroups with and without T2DM

|  | With T2DM (n = 721) | | | Without T2DM (n = 1386) | | |
| --- | --- | --- | --- | --- | --- | --- |
|  | MACCE  (n = 89) | Non-MACCE  (n = 632) | P-value | MACCE  (n = 98) | Non-MACCE  (n = 1288) | P-value |
| IR surrogates |  |  |  |  |  |  |
| TyG index | 9.58 ± 0.63 | 9.07 ± 0.63 | < 0.001 | 9.04 ± 0.48 | 8.70 ± 0.51 | < 0.001 |
| VAI | 4.37 ± 2.56 | 3.10 ± 2.21 | < 0.001 | 3.57 ± 2.47 | 2.55 ± 1.66 | < 0.001 |
| CVAI | 163.05 ± 46.55 | 144.69 ± 43.57 | < 0.001 | 140.77 ± 41.71 | 123.26 ± 43.42 | < 0.001 |
| LAP | 88.33 ± 57.60 | 57.82 ± 44.79 | < 0.001 | 65.39 ± 52.42 | 44.05 ± 32.47 | < 0.001 |
| TG/HDL-C | 6.09 ± 3.56 | 4.57 ± 3.04 | < 0.001 | 5.42 ± 3.44 | 4.03 ± 2.58 | < 0.001 |
| Age, years | 60.03 ± 9.32 | 60.93 ± 8.17 | 0.391 | 62.36 ± 10.31 | 59.39 ± 9.24 | 0.002 |
| Gender, female, n (%) | 37 (41.6) | 190 (30.1) | 0.029 | 28 (28.6) | 336 (26.1) | 0.590 |
| BMI, kg/m^2^ | 27.29 ± 3.36 | 26.60 ± 3.19 | 0.057 | 25.65 ± 3.05 | 25.78 ± 3.17 | 0.711 |
| WC, cm | 98.75 ± 13.01 | 94.40 ± 12.29 | 0.002 | 92.04 ± 12.33 | 89.35 ± 11.91 | 0.032 |
| Heart rate, bpm | 73.16 ± 10.93 | 71.60 ± 9.99 | 0.174 | 69.09 ± 9.54 | 68.73 ± 10.05 | 0.735 |
| SBP, mmHg | 131.02 ± 17.59 | 131.92 ± 17.19 | 0.647 | 128.68 ± 18.28 | 129.37 ± 15.83 | 0.715 |
| DBP, mmHg | 76.68 ± 9.58 | 76.72 ± 10.31 | 0.971 | 75.38 ± 10.86 | 77.19 ± 9.43 | 0.070 |
| Smoking history, n (%) | 47 (52.8) | 336 (53.2) | 0.950 | 56 (57.1) | 756 (58.7) | 0.764 |
| Drinking history, n (%) | 20 (22.5) | 155 (24.5) | 0.672 | 24 (24.5) | 296 (23.0) | 0.733 |
| Family history of CAD, n (%) | 7 (7.9) | 78 (12.3) | 0.220 | 7 (7.1) | 126 (9.8) | 0.392 |
| Medical history, n (%) |  |  |  |  |  |  |
| T2DM | 89 (100.0) | 632 (100.0) | 1.000 | - | - | - |
| Duration of diabetes, years | 8.57 ± 4.19 | 8.14 ± 4.30 | 0.375 | - | - | - |
| Hypertension | 65 (73.0) | 451 (71.4) | 0.743 | 64 (65.3) | 725 (56.3) | 0.082 |
| Previous MI | 30 (33.7) | 130 (20.6) | 0.005 | 37 (37.8) | 243 (18.9) | < 0.001 |
| Previous PCI | 25 (28.1) | 115 (18.2) | 0.027 | 20 (20.4) | 199 (15.5) | 0.195 |
| Previous stroke | 11 (12.4) | 87 (13.8) | 0.717 | 20 (20.4) | 117 (9.1) | < 0.001 |
| Previous PAD | 1 (1.1) | 22 (3.5) | 0.388 | 5 (5.1) | 45 (3.5) | 0.410 |
| Clinical diagnosis, n (%) |  |  | 0.131 |  |  | 0.124 |
| NSTEMI | 22 (24.7) | 114 (18.0) |  | 21 (21.4) | 200 (15.5) |  |
| UA | 67 (75.3) | 518 (82.0) |  | 77 (78.6) | 1088 (84.5) |  |
| Laboratory tests |  |  |  |  |  |  |
| TG, mmol/L | 2.32 ± 1.06 | 1.74 ± 0.95 | < 0.001 | 2.10 ± 1.07 | 1.62 ± 0.82 | < 0.001 |
| TC, mmol/L | 4.37 ± 1.19 | 4.02 ± 1.01 | 0.003 | 4.39 ± 1.05 | 4.21 ± 1.04 | 0.100 |
| LDL-C, mmol/L | 2.57 ± 0.94 | 2.41 ± 0.85 | 0.101 | 2.63 ± 0.87 | 2.57 ± 0.89 | 0.513 |
| HDL-C, mmol/L | 0.94 ± 0.22 | 0.95 ± 0.23 | 0.592 | 0.96 ± 0.22 | 1.01 ± 0.24 | 0.054 |
| hs-CRP, mg/L | 1.80 (0.89, 4.54) | 1.61 (0.68, 3.95) | 0.201 | 1.06 (0.53, 3.90) | 1.16 (0.51, 2.76) | 0.709 |
| Creatinine, μmol/L | 72.44 ± 17.01 | 73.18 ± 16.71 | 0.695 | 77.90 ± 14.31 | 77.26 ± 16.52 | 0.706 |
| eGFR, mL/min/1.73 m^2^ | 95.41 ± 21.42 | 96.99 ± 21.37 | 0.516 | 89.33 ± 19.08 | 92.16 ± 18.96 | 0.154 |
| Uric acid, μmol/L | 332.48 ± 80.22 | 327.45 ± 74.86 | 0.557 | 368.12 ± 80.72 | 352.51 ± 82.16 | 0.070 |
| FBG, mmol/L | 9.16 ± 3.62 | 7.41 ± 2.18 | < 0.001 | 5.68 ± 0.69 | 5.29 ± 0.58 | < 0.001 |
| HbA1c, % | 8.18 ± 1.18 | 7.33 ± 1.29 | < 0.001 | 5.74 ± 0.35 | 5.64 ± 0.39 | 0.010 |
| LVEF, % | 63.42 ± 6.66 | 63.91 ± 6.60 | 0.509 | 61.73 ± 9.08 | 64.28 ± 6.49 | 0.007 |
| Medications at admission, n (%) |  |  |  |  |  |  |
| ACEI/ARB | 24 (27.0) | 162 (25.6) | 0.788 | 22 (22.4) | 262 (20.3) | 0.618 |
| DAPT | 26 (29.2) | 208 (32.9) | 0.485 | 32 (32.7) | 366 (28.4) | 0.371 |
| Aspirin | 50 (56.2) | 341 (54.0) | 0.693 | 53 (54.1) | 661 (51.3) | 0.598 |
| P2Y12 inhibitors | 27 (30.3) | 218 (34.5) | 0.438 | 37 (37.8) | 390 (30.3) | 0.122 |
| β-blocker | 19 (21.3) | 134 (21.2) | 0.975 | 32 (32.7) | 283 (22.0) | 0.015 |
| Statins | 28 (31.5) | 185 (29.3) | 0.672 | 36 (36.7) | 400 (31.1) | 0.243 |
| Oral antidiabetic agents | 48 (53.9) | 327 (51.7) | 0.698 | - | - | - |
| Insulin | 29 (32.6) | 169 (26.7) | 0.247 | - | - | - |
| Medications at discharge, n (%) |  |  |  |  |  |  |
| ACEI/ARB | 77 (86.5) | 480 (75.9) | 0.026 | 77 (78.6) | 821 (63.7) | 0.003 |
| DAPT | 89 (100.0) | 631 (99.8) | > 0.999 | 98 (100.0) | 1288 (100.0) | 1.000 |
| Aspirin | 89 (100.0) | 631 (99.8) | > 0.999 | 98 (100.0) | 1288 (100.0) | 1.000 |
| P2Y12 inhibitors | 89 (100.0) | 632 (100.0) | 1.000 | 98 (100.0) | 1288 (100.0) | 1.000 |
| β-blocker | 81 (91.0) | 591 (93.5) | 0.380 | 90 (91.8) | 1148 (89.1) | 0.403 |
| Statins | 88 (98.9) | 624 (98.7) | > 0.999 | 95 (96.9) | 1258 (97.7) | 0.909 |
| Oral antidiabetic agents | 47 (52.8) | 325 (51.4) | 0.807 | - | - | - |
| Insulin | 27 (30.3) | 163 (25.8) | 0.362 | - | - | - |
| Coronary procedural information |  |  |  |  |  |  |
| LM disease, n (%) | 10 (11.2) | 30 (4.7) | 0.012 | 8 (8.2) | 45 (3.5) | 0.020 |
| Three-vessel disease, n (%) | 51 (57.3) | 265 (41.9) | 0.006 | 26 (26.5) | 289 (22.4) | 0.351 |
| Chronic total occlusion, n (%) | 23 (25.8) | 90 (14.2) | 0.005 | 16 (16.3) | 148 (11.5) | 0.153 |
| Diffuse lesion, n (%) | 33 (37.1) | 179 (28.3) | 0.090 | 22 (22.4) | 274 (21.3) | 0.784 |
| Bifurcation lesion, n (%) | 24 (27.0) | 143 (22.6) | 0.364 | 16 (16.3) | 252 (19.6) | 0.434 |
| SYNTAX score | 14.44 ± 6.58 | 11.75 ± 5.32 | < 0.001 | 11.39 ± 5.54 | 9.75 ± 5.21 | 0.003 |
| Target vessel territory, n (%) |  |  |  |  |  |  |
| LM | 4 (4.5) | 17 (2.7) | 0.541 | 4 (4.1) | 27 (2.1) | 0.354 |
| LAD | 55 (61.8) | 405 (64.1) | 0.675 | 60 (61.2) | 859 (66.7) | 0.270 |
| LCX | 35 (39.3) | 267 (42.2) | 0.601 | 26 (26.5) | 396 (30.7) | 0.382 |
| RCA | 50 (56.2) | 314 (49.7) | 0.251 | 40 (40.8) | 491 (38.1) | 0.597 |
| Complete revascularization, n (%) | 38 (42.7) | 330 (52.2) | 0.093 | 52 (53.1) | 817 (63.4) | 0.041 |
| Number of stents | 2.25 ± 1.51 | 2.10 ± 1.26 | 0.376 | 1.94 ± 1.16 | 1.91 ± 1.26 | 0.850 |

*IR* insulin resistance, *TyG* triglyceride-glucose, *VAI* visceral adiposity index, *CVAI* Chinese visceral adiposity index, *LAP* lipid accumulation product, *TG/HDL-C* triglyceride to high-density lipoprotein cholesterol ratio, *BMI* body mass index, *WC* waist circumference, *SBP* systolic blood pressure, *DBP* diastolic blood pressure, *CAD* coronary artery disease, *T2DM* type 2 diabetes mellitus, *MI* myocardial infarction, *PCI* percutaneous coronary intervention, *PAD* peripheral artery disease, *NSTEMI* non-ST-segment elevation myocardial infarction, *UA* unstable angina, *TG* triglyceride, *TC* total cholesterol, *LDL-C* low-density lipoprotein cholesterol, *HDL-C* high-density lipoprotein cholesterol, *hs-CRP* high-sensitivity C-reactive protein, *eGFR* estimated glomerular filtration rate, *FBG* fasting blood glucose, *HbA1c* glycosylated hemoglobin A1c, *LVEF* left ventricular ejection fraction, *ACEI* angiotensin converting enzyme inhibitor, *ARB* angiotensin receptor blocker, *DAPT* dual antiplatelet therapy, *LM* left main artery, *SYNTAX* synergy between PCI with taxus and cardiac surgery, *LAD* left anterior descending artery, *LCX* left circumflex artery, *RCA* right coronary artery, *MACCE* major adverse cardiac and cerebrovascular events

# Table S3. Predictive value of various IR surrogates for the risk of MACCE in subgroups with and without T2DM

| IR surrogates | Variate type | Subgroups | No. MACCE  Lower/Higher | Unadjusted analysis | | | Adjusted analysis^c^ | | | |
| --- | --- | --- | --- | --- | --- | --- | --- | --- | --- | --- |
|  |  |  |  | HR (95% CI) | P-value | P for interaction | HR (95% CI) | P-value | P for interaction |  |
| TyG index | Nominal^a^ | T2DM (+) | 14/75 | 5.910 (3.340-10.459) | < 0.001 | 0.541 | 4.130 (2.247-7.589) | < 0.001 | 0.892 |  |
|  |  | T2DM (−) | 18/80 | 4.695 (2.816-7.830) | < 0.001 |  | 4.688 (2.765-7.950) | < 0.001 |  |  |
|  | Continuous^b^ | T2DM (+) | - | 2.109 (1.707-2.607) | < 0.001 | 0.513 | 1.759 (1.375-2.250) | < 0.001 | 0.733 |  |
|  |  | T2DM (−) | - | 1.924 (1.569-2.359) | < 0.001 |  | 1.945 (1.567-2.415) | < 0.001 |  |  |
| VAI | Nominal^a^ | T2DM (+) | 24/65 | 2.850 (1.785-4.552) | < 0.001 | 0.448 | 2.160 (1.332-3.501) | 0.002 | 0.897 |  |
|  |  | T2DM (−) | 31/67 | 2.244 (1.466-3.434) | < 0.001 |  | 2.133 (1.380-3.299) | 0.001 |  |  |
|  | Continuous^b^ | T2DM (+) | - | 1.463 (1.252-1.708) | < 0.001 | 0.758 | 1.362 (1.143-1.624) | 0.001 | 0.510 |  |
|  |  | T2DM (−) | - | 1.521 (1.322-1.751) | < 0.001 |  | 1.495 (1.299-1.721) | < 0.001 |  |  |
| CVAI | Nominal^a^ | T2DM (+) | 32/57 | 1.866 (1.210-2.877) | 0.005 | 0.790 | 1.599 (1.024-2.497) | 0.039 | 0.872 |  |
|  |  | T2DM (−) | 33/65 | 2.034 (1.338-3.093) | 0.001 |  | 1.699 (1.093-2.639) | 0.018 |  |  |
|  | Continuous^b^ | T2DM (+) | - | 1.456 (1.195-1.775) | < 0.001 | 0.959 | 1.400 (1.128-1.737) | 0.002 | 0.675 |  |
|  |  | T2DM (−) | - | 1.449 (1.201-1.749) | < 0.001 |  | 1.369 (1.119-1.676) | 0.002 |  |  |
| LAP | Nominal^a^ | T2DM (+) | 23/66 | 3.061 (1.904-4.920) | < 0.001 | 0.261 | 2.468 (1.507-4.044) | < 0.001 | 0.297 |  |
|  |  | T2DM (−) | 32/66 | 2.140 (1.403-3.264) | < 0.001 |  | 1.901 (1.230-2.939) | 0.004 |  |  |
|  | Continuous^b^ | T2DM (+) | - | 1.508 (1.306-1.741) | < 0.001 | 0.903 | 1.438 (1.219-1.695) | < 0.001 | 0.999 |  |
|  |  | T2DM (−) | - | 1.537 (1.343-1.759) | < 0.001 |  | 1.524 (1.314-1.766) | < 0.001 |  |  |
| TG/HDL-C | Nominal^a^ | T2DM (+) | 26/63 | 2.551 (1.615-4.029) | < 0.001 | 0.310 | 2.030 (1.261-3.269) | 0.004 | 0.522 |  |
|  |  | T2DM (−) | 35/63 | 1.864 (1.233-2.817) | 0.003 |  | 1.697 (1.111-2.593) | 0.015 |  |  |
|  | Continuous^b^ | T2DM (+) | - | 1.412 (1.203-1.657) | < 0.001 | 0.750 | 1.322 (1.104-1.583) | 0.002 | 0.355 |  |
|  |  | T2DM (−) | - | 1.468 (1.267-1.701) | < 0.001 |  | 1.518 (1.296-1.778) | < 0.001 |  |  |

*IR* insulin resistance, *TyG* triglyceride-glucose, *VAI* visceral adiposity index, *CVAI* Chinese visceral adiposity index, *LAP* lipid accumulation product, *TG/HDL-C* triglyceride-to-high density lipoprotein cholesterol ratio, *T2DM* type 2 diabetes mellitus, *MACCE* major adverse cardiac and cerebrovascular events, *HR* hazard ratio, *CI* confidence interval

^a^ The HR was examined by regarding the lower median as reference

^b^ The HR was examined by evaluating 1 normalized unit increase

^c^ Adjusted for smoking history, hypertension, previous MI, previous PCI, previous stroke, clinical diagnosis, TC, hs-CRP, eGFR, HbA1c, LVEF, ACEI/ARB at discharge, oral antidiabetic agents at discharge, insulin at discharge, LM disease, three-vessel disease, chronic total occlusion, SYNTAX score, complete revascularization, and number of stents

# Table S4. Diagnostic performance of IR surrogates for MACCE in subgroups with and without T2DM

|  | AUC | | | Cut-off value | Sensitivity, % | Specificity, % |
| --- | --- | --- | --- | --- | --- | --- |
|  | Est. (95% CI) | P-value | P for comparison |  |  |  |
| With T2DM |  |  |  |  |  |  |
| TyG index | 0.719 (0.684-0.751) | < 0.001 | - | 9.18 | 82.02 | 59.18 |
| VAI | 0.677 (0.641-0.711) | < 0.001 | 0.035 | 2.34 | 80.90 | 47.94 |
| CVAI | 0.615 (0.579-0.651) | < 0.001 | 0.002 | 153.70 | 61.80 | 61.71 |
| LAP | 0.686 (0.651-0.720) | < 0.001 | 0.105 | 60.87 | 65.17 | 67.72 |
| TG/HDL-C | 0.652 (0.616-0.687) | < 0.001 | 0.001 | 4.11 | 70.79 | 57.28 |
| Without T2DM |  |  |  |  |  |  |
| TyG index | 0.687 (0.662-0.712) | < 0.001 | - | 8.85 | 75.51 | 61.02 |
| VAI | 0.651 (0.625-0.676) | < 0.001 | 0.013 | 1.96 | 78.57 | 45.81 |
| CVAI | 0.616 (0.590-0.642) | < 0.001 | 0.026 | 117.38 | 76.53 | 47.59 |
| LAP | 0.637 (0.611-0.663) | < 0.001 | 0.011 | 23.32 | 88.78 | 31.91 |
| TG/HDL-C | 0.636 (0.610-0.662) | < 0.001 | < 0.001 | 3.02 | 78.57 | 43.25 |

*T2DM* type 2 diabetes mellitus, *TyG* triglyceride-glucose, *VAI* visceral adiposity index, *CVAI* Chinese visceral adiposity index, *LAP* lipid accumulation product, *TG/HDL-C* triglyceride-to-high density lipoprotein cholesterol ratio, *AUC* area under the ROC curve, *CI* confidence interval

# Table S5. Incremental ability of various IR surrogates on the prediction of MACCE in subgroups with and without T2DM

|  | Harrell’s C-index | | | Continuous NRI | | IDI | |
| --- | --- | --- | --- | --- | --- | --- | --- |
|  | Est. (95% CI) | ΔEst. | P-value | Est. (95% CI) | P-value | Est. (95% CI) | P-value |
| With T2DM |  |  |  |  |  |  |  |
| Baseline model^a^ | 0.712 (0.657-0.768) | - | - | - | - | - | - |
| + TyG index | 0.756 (0.705-0.806) | 0.044 | 0.005 | 0.241 (0.057-0.350) | 0.007 | 0.037 (0.005-0.079) | 0.007 |
| + VAI | 0.733 (0.680-0.787) | 0.021 | 0.063 | 0.149 (0.020-0.290) | 0.040 | 0.024 (0.002-0.067) | 0.033 |
| + CVAI | 0.740 (0.688-0.792) | 0.028 | 0.029 | 0.127 (−0.027-0.272) | 0.086 | 0.013 (−0.003-0.062) | 0.206 |
| + LAP | 0.753 (0.702-0.804) | 0.041 | 0.004 | 0.187 (0.036-0.324) | < 0.001 | 0.026 (0.000-0.072) | 0.047 |
| + TG/HDL-C | 0.729 (0.675-0.783) | 0.017 | 0.120 | 0.135 (−0.037-0.242) | 0.073 | 0.018 (−0.001-0.062) | 0.066 |
| Without T2DM |  |  |  |  |  |  |  |
| Baseline model^a^ | 0.701 (0.651-0.751) | - | - | - | - | - | - |
| + TyG index | 0.745 (0.697-0.793) | 0.044 | 0.010 | 0.284 (0.165-0.396) | < 0.001 | 0.049 (0.015-0.107) | < 0.001 |
| + VAI | 0.718 (0.667-0.769) | 0.017 | 0.134 | 0.163 (0.040-0.256) | < 0.001 | 0.031 (0.012-0.071) | < 0.001 |
| + CVAI | 0.710 (0.659-0.761) | 0.009 | 0.399 | 0.114 (−0.004-0.232) | 0.060 | 0.010 (0.001-0.039) | 0.013 |
| + LAP | 0.712 (0.659-0.765) | 0.011 | 0.317 | 0.177 (0.041-0.292) | 0.007 | 0.046 (0.013-0.099) | < 0.001 |
| + TG/HDL-C | 0.718 (0.667-0.768) | 0.017 | 0.145 | 0.183 (0.052-0.295) | 0.007 | 0.033 (0.009-0.077) | < 0.001 |

*T2DM* type 2 diabetes mellitus, *TyG* triglyceride-glucose, *VAI* visceral adiposity index, *CVAI* Chinese visceral adiposity index, *LAP* lipid accumulation product, *TG/HDL-C* triglyceride-to-high density lipoprotein cholesterol ratio, *NRI* net reclassification improvement, *IDI* integrated discrimination improvement, *CI* confidence interval

^a^ The baseline model incorporates smoking history, hypertension, previous MI, previous PCI, previous stroke, TC, eGFR, HbA1c, LVEF, LM disease, three-vessel disease, SYNTAX score, and number of stents
