# Supplementary material for: Comparison of various insulin resistance surrogates on prognostic prediction and stratification following percutaneous coronary intervention in patients with and without type 2 diabetes mellitus
Source: Cardiovasc Diabetol. 2021 Sep 18;20:190. doi: 10.1186/s12933-021-01383-7 (PMC8449896; doi:10.1186/s12933-021-01383-7)
Supplement: Supplementary file 2 — Additional file 2. Additional Figures. [file 12933_2021_1383_MOESM2_ESM.docx]

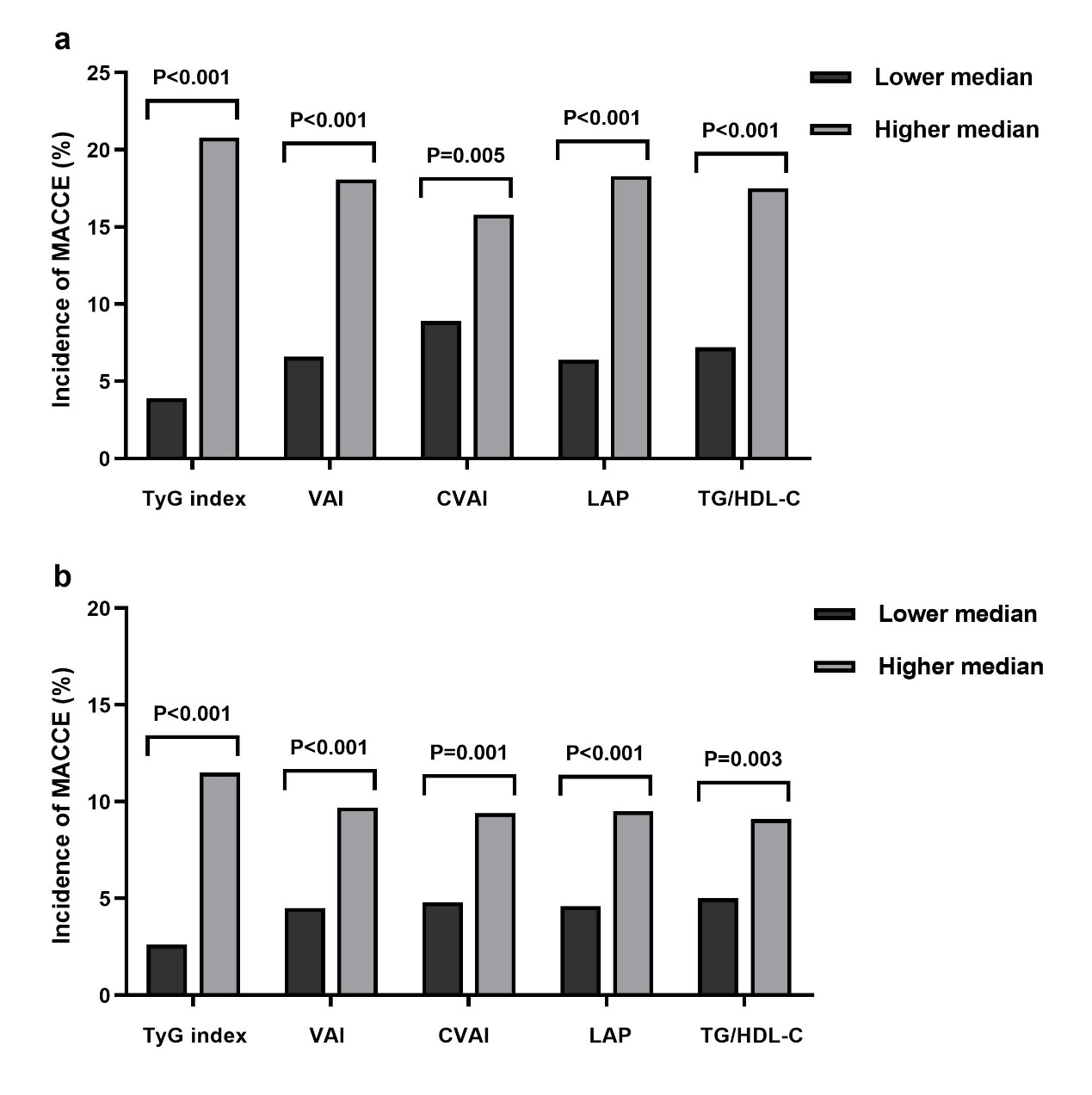


Figure S1. Incidence of MACCE according to the median of respective IR surrogates in subgroups with T2DM (a) and without T2DM (b).

*MACCE* major adverse cardiac and cerebrovascular events, *TyG* triglyceride-glucose, *VAI* visceral adiposity index, *CVAI* Chinese visceral adiposity index, *LAP* lipid accumulation product, *TG/HDL-C* triglyceride-to-high density lipoprotein cholesterol ratio


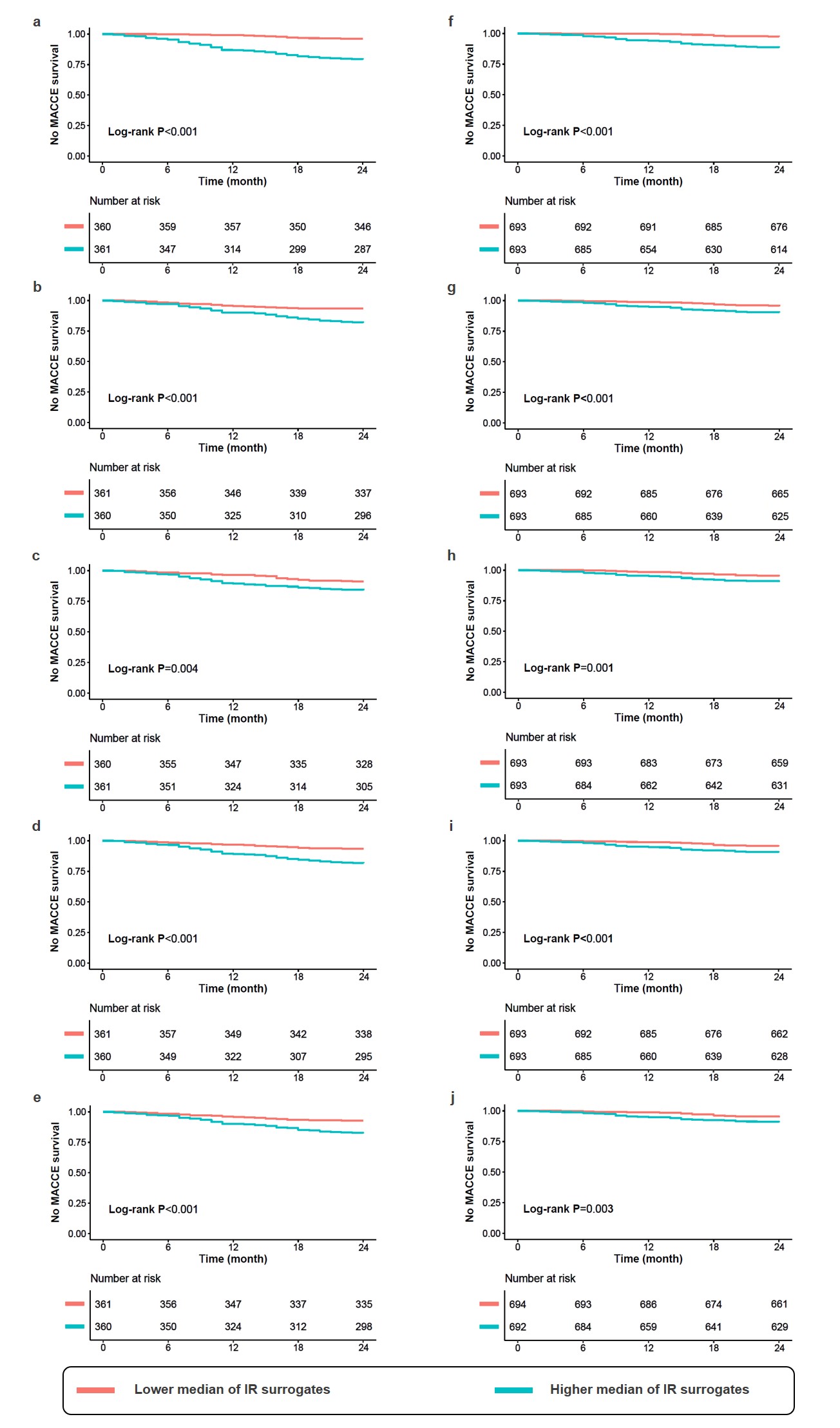


Figure S2. Kaplan-Meier curves for no MACCE survival according to the median of TyG index (a, f), VAI (b, g), CVAI (c, h), LAP (d, i), and TG/HDL-C (e, j) in subgroups with T2DM (a-e) and without T2DM (f-j).

*MACCE* major adverse cardiac and cerebrovascular events, *IR* insulin resistance


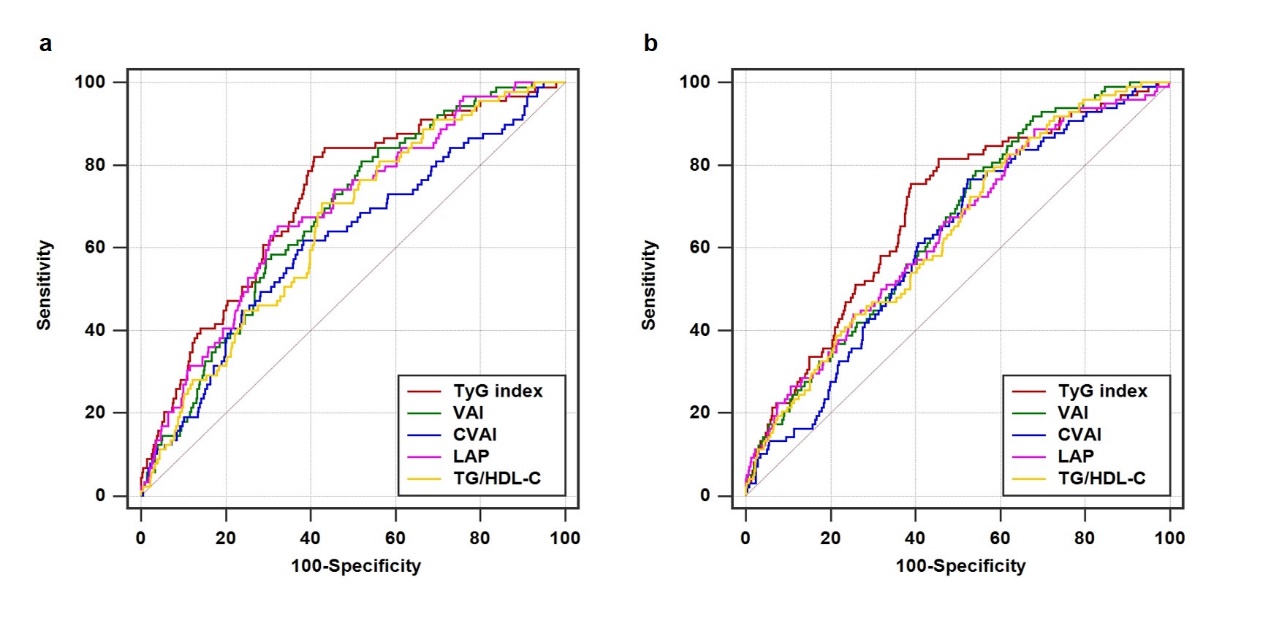


Figure S3. ROC curves evaluating the diagnostic performance of each IR surrogate for MACCE in subgroups with T2DM (a) and without T2DM (b).

*TyG* triglyceride-glucose, *VAI* visceral adiposity index, *CVAI* Chinese visceral adiposity index, *LAP* lipid accumulation product, *TG/HDL-C* triglyceride-to-high density lipoprotein cholesterol ratio
